# Shear damage mechanisms of jointed rock mass: a macroscopic and mesoscopic study

**DOI:** 10.1038/s41598-024-59281-3

**Published:** 2024-04-14

**Authors:** Gang Wang, Wenhao Liu, Feng Jiang, Peng He, Na Huang, Zhiyong Xiao, Chengcheng Zheng

**Affiliations:** 1 Shandong University of Science and Technology, Qingdao, 266590 China; 2 College of Civil Engineering, Fujian University of Technology, Fuzhou, 350118 China

**Keywords:** Cohesive element, Shearing process, Peak strength, Failure type, Civil engineering, Solid Earth sciences

## Abstract

The joints are existing throughout the underground rock mass. It is of great significance to investigate the shear performance of the rock mass to maintain the stability of the underground structure. In this study, we conducted orthogonal tests to determine the proportion of rock-like materials, and used JRC curves to make specimen molds and then prepare the specimens. We conducted straight shear tests and uniaxial compression tests to determine the various mechanical parameters of the rock-like materials. Next, we carried out the compression and shear tests to investigate the shear characteristics of the specimens, and study the damage pattern and shear strength of the jointed rock mass under different confining pressures and roughness levels. The mesoscopic displacements in the shear process of joints were analyzed by using ABAQUS. The test results show that the effect of the confining pressure on the shear strength of the joint plane is relatively obvious, and a larger confining pressure indicates a larger shear strength. The effects of different joint plane roughness and shear rated on the shear characteristics of the joint plane are also significant. The mesoscopic displacement difference inside the joint plane with higher roughness is relatively large, and the stress concentration phenomenon is obvious and lasts longer, which leads to the faster destruction of the specimen with higher roughness and the higher destruction degree. Therefore, we suggest that the priority should be given to the reinforcement of jointed rock mass with high roughness during the construction to prevent sudden destabilization and failure.

## Introduction

**Figure 1:**
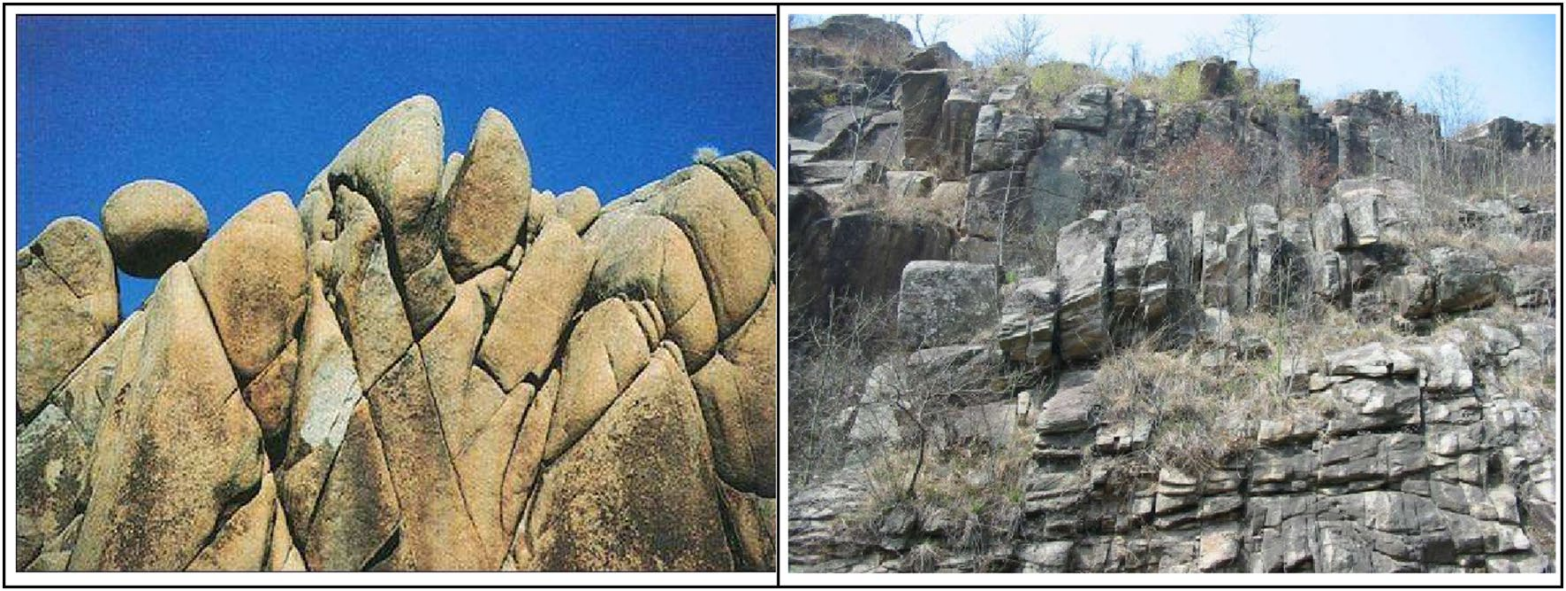
Jointed rock mass in nature.

The natural rock mass consists of joints and blocks divided by them. The joints can damage the integrity of the rock mass, and reduce its mechanical properties. The complexity and variety of the rock’s existence as the geological formations have changed over time and led to the prevalence of laminated rock joints in the rock structure (see Fig. 1). The great uncertainty in the force state of the joint plane and the number of joints of rock mass leads to the destabilization and failure of rock mass in many forms. Therefore, it is essential to investigate the shear strength and deformation failure mechanism of jointed rock mass for project construction.

Natural jointed rock mass has been a problem that affects the accuracy of the studies of their shear mechanisms due to the complex and random undulation of the jointed planes. During the geological formation for a long time, the surface morphology of jointed rock mass features great randomness. And there are two types according to the scale of undulation on the joint plane: undulation (a larger scale), and roughness (for random concave convex surfaces)^1,2^. The undulating surfaces of joints are often nested with each other, and when the normal load is small, the joints will slip and heave sideways due to the presence of the undulation, thus the dislocation and movement of the joints are mainly caused by the undulating surfaces. And under the action of straight shear, the roughness surface is firstly damaged by abrasion. Roughness and undulation constitute the surface characteristics of joint plane, and the mutual combination of various random roughnesses and their amplitudes results in the complex layered rock jointed planes in nature. When scholars consider the surface characteristics of joint planes, they often assume some relatively ideal and uniform surface morphology of joint planes for related research^3^.

The research related to the analysis of straight shear damage mechanism of the joint plane has been popular among scholars across the globe. In the shear process of the joint plane, factors such as the surface morphology of the joint plane, the normal load value and the shear rate all have an effect on the peak shear intensity^4–8^. When subjected to lower normal loads, the jointed rock mass climbs along the joint undulation surface^9^. As the climbing phenomenon occurs, the surface of the joint plane produces slip wear damage and the frictional strength of the joint plane is increased. When the normal load is larger, the shear expansion phenomenon of the jointed rock mass gradually becomes smaller, and the rock mass climbing phenomenon gradually disappears. Under the action of horizontal load, the small protrusions on the joint plane undergo shearing damage. When the normal load is small, the specimen is mainly dominated by the abrasion damage of the roughness surface of laminated rock joints. When the normal load is larger, the climbing phenomenon is not obvious, and the shear damage of the specimen at this time is mainly dominated by the shear fracture damage of the undulating surface of the rock joints. Therefore, the higher the normal load, the higher the shear strength in the case of different normal loads^10^.

Due to the uneconomical nature of experimental studies on natural rock joints, and the non-reusability of specimens, many scholars both domestically and internationally have turned to numerical simulation for related research^11–14^. With the prevalence of numerous joints in rocks, the discrete element method based on discontinuous media mechanics has been increasingly employed in recent years to simulate the rock fracture process^15–17^. However, rocks are not entirely discontinuous media, and traditional finite element methods overlook the discreteness of rocks. In consideration of this, this paper adopts a novel computational approach developed by ABAQUS Corporation, which analyzes the computational objects through the bulk embedding of zero-thickness cohesive elements. Unlike traditional finite elements, this finite element cohesive element is more focused on simulating the microstructure of materials and can intuitively demonstrate the discontinuity of rock materials. This approach discretizes the finite element body through a mesh, enabling both the mechanistic developmental changes of the continuous medium before failure and the failure of the continuous body through cohesive element failure, effectively avoiding the singularity problem at the crack tip during rock mass failure. Currently, this method has been utilized by scholars in various fields in recent years and has achieved satisfactory results^18,19^. Additionally, scholars have conducted in-depth research on the problems encountered in rock engineering through finite element methods^20–24^, discrete element methods^25–29^, and boundary element methods^30–34^.

Even if the rock mass is segmented by joints, there are still many continuous media in the segmented rock mass, and the discrete element software is unable to simulate the mechanical properties of the jointed rock mass by discretizing it. The basic principle of discrete element is to set a certain number of round particles. However, due to the limitation of computing equipment, the sizes of natural rock particles are often different by several orders of magnitude, which has a great influence on the experimental accuracy. In addition, non-deformable rigid particles are a good way to simulate natural rock particles. The current commercial calculation software and simulation methods are not enough to reflect the various mechanical relationships within the jointed rock mass, and the calculation models and methods are not yet able to effectively reflect the interactions between the jointed rock mass, and it is difficult to accurately analyze and evaluate the geologic conditions, so that the stability evaluation of the jointed rock mass remains in the empirical or semi-empirical and semi-theoretical stage. As people have not yet fully understood the interaction mechanism between joints, especially the localized failure mechanism of jointed rock mass near the structural plane and the reinforcement of structural plane under long-term complicated geological conditions, the existing research results have not been able to fully reveal the interaction mechanism of structural surfaces under complex geological conditions, which greatly limits the fine development of geotechnical engineering and scientific construction.

Therefore, in this work, through the compression shear tests on rock-like material specimens in lab, we study the shear performance of jointed rock specimens under different shear conditions. On the ABAQUS platform, the compression shear process of jointed rock mass with different roughness is taken as the research object, and the compression shear test model of jointed rock mass is established by selecting globally embedded zero-thickness viscous cohesive unit, which reproduces the shear process of the jointed rock mass in a lifelike way. By comparing the numerical simulation with the joint plane shear test, the correctness of this model is verified and its superiority is explored, and then the numerical model of jointed rock mass is established. By intercepting the displacement cloud map in the numerical simulation of the shear process, we investigate the shear process of the jointed rock mass, and explore the destructive mechanism.

## Relative works

In natural rock masses, numerous randomly distributed and varying-sized joints significantly influence the mechanical properties of the rock. In recent years, many scholars have conducted many tests to investigate the mechanical behavior and damage mechanisms of jointed rock mass^35–39^. Among these studies, the shear performance of jointed rock masses has been identified as a crucial indicator for analyzing engineering stability, drawing extensive attention^40–43^.

### Cutting-edge results on the relationship between roughness and shear characteristics of jointed rock mass

The influence of joint roughness on the jointed rock mass mechanical properties is particularly significant. In order to quantify the joint plane roughness, Barton^44^ proposed the JRC-JSC shear strength model that considers joint plane roughness and provided 10 standard joint profiles. Despite recent advancements, including the proposal of a new joint contact state coefficient for quantifying three-dimensional roughness^45^, characterization of joint surface roughness at the microscopic scale through photogrammetry^46^, and the introduction of the FCE method for evaluating joint plane roughness considering various morphological parameters^47^, JRC-based roughness quantification remains concise and effective. Based on JRC to characterize roughness, studies on the rock mass mechanical properties, such as that by Han et al.^48^, have investigated the influence of surface roughness represented by joint roughness coefficient on shear stress, normal stress, evolution of normal displacement, and failure modes. It was concluded that under constant normal stress boundary conditions, with increasing JRC values, the peak shear stress of a single joint specimen increases significantly, and the peak normal displacement of a double-jointed specimen increases significantly. In jointed rock masses with anchor bolts, JRC’s impact on shear performance is also noteworthy. Wang et al.^49^ explored the shear mechanical performance characteristics and failure mechanisms of anchored nodes under different surface roughness and anchoring conditions. Li et al.^50^ investigated the influence of JRC and anchor angle on the mechanical performance of anchored structural surfaces, concluding a positive correlation between peak shear strength, residual strength, and JRC. The fluctuating shape of the structural surface leads to a noticeable decline in the strain softening stage of the structural surface. Wu et al.^51^ studied the effects of cyclic shear loading on jointed rock masses under different joint roughness conditions, finding that an increase in JRC significantly raises the peak shear stress and normal displacement for both direct shear and the first cycle. However, these studies have not fully revealed the relationship between roughness and fracture distribution, and whether roughness is equally influenced by changes in other shear conditions affecting shear performance.

### Cutting-edge results on the relationship between loading conditions and joint shear characteristics

The mechanical performance of jointed rock mass is closely associated with the loading conditions, and the influence of shear rate on shear performance has been a topic of extensive research in laboratory tests. He et al.^52^ conducted constant normal direct shear tests on jointed specimens at different shear rates, and investigated the shear performance with different joint roughness coefficients and joint wall compressive strengths, and under different normal stresses. Meng et al.^53^ and Jiang et al.^54^ conducted direct shear tests under various shear rate conditions to investigate the influence of normal stiffness and shear rate on the mechanical properties of the material. The results indicated that with an increase in shear rate, the peak shear stress of the joint decreased nonlinearly, while the peak shear displacement increased. Additionally, more local cracks appeared on the joint surface, and the joint dilation slightly decreased. However, the joint shear strength estimated under low shear rates in the laboratory cannot be applied to field conditions. To study the impact of high shear rates on shear performance, Tiwari^55^ developed a probabilistic method to estimate the in-situ shear strength of joints under high displacement rate conditions. That study found that the shear strength of structural plane decreases with increasing shear rate, and this dependence is more significant under low-density rock and high confining stress conditions. Confining pressure also affects the shear performance of jointed rock masses. Chen et al.^56^, Zhu et al.^57^, and others investigated the variations in mechanical characteristics and strength features of non-persistent jointed rock masses under different confining pressures through direct shear tests. It was concluded that the residual shear strength of jointed rock masses increases with increasing confining pressure, and confining pressure significantly affected the peak shear strength and residual strength of jointed rock mass with the same roughness on both sides of the joint. The aforementioned scholars have individually conducted detailed studies on the influence of shear rate and confining pressure on the performance of jointed rock masses. However, integrating these two factors when studying the performance of jointed rock masses and investigating whether loading conditions are affected by changes in other shear conditions remain areas for further research.

### Cutting-edge results of numerical simulation applications in the study of joint shear characteristics

It is difficult to observe internal changes of shear failure mechanism of joint plane in laboratory experiments. Numerical simulation, as a research method, provides a comprehensive analysis of macroscopic and microscopic changes in joint plane. Scholars have utilized Discrete Element Method (DEM) software, particularly Particle Flow Code (PFC), for simulations to investigate the internal damage of rocks. Huan et al.^58^ employed PFC to conduct constant normal stress direct shear tests on joint specimens and studied the local failure modes during the shear process. Bahaaddini^59^ used PFC to explore the impact of rock joint roughness and continuity on the mechanical behavior of rock structures. Meng er al.^60^ simulated the failure mechanisms and damage evolution of hard rock joints using software, revealing that rougher joints produced more cracks under higher normal stress. In the rapid growth stage of shear under high normal stress, cracks in rough joints expand faster. As rocks are continuous materials, Finite Element Method (FEM) software is also applicable for studying the mechanical properties and failure mechanisms of rocks. Liu et al.^61^ used the FLAC3D finite difference software to simulate the rock failure process and investigated the influence of microscopic cracks on rock behavior. Han et al.^62^ established a corresponding numerical model by inserting zero-thickness cohesive elements into the finite element model. That study focused on the shear characteristics of rock materials with crack-like voids. The results indicated that from the perspective of cohesive elements, the shear process of rock materials with crack-like voids can generally be divided into four typical stages: elastic strengthening, crack strengthening, plastic softening, and residual strength.

While many scholars have used experimental and numerical simulation methods to reveal the macroscopic aspects of the shear performance of jointed rock mass, there is insufficient research from a microscopic perspective. Few attention is paid to the distribution of random cracks during joint shear processes and microscopic damage around joints. Therefore, further in-depth studies are needed to investigate the mechanisms of failure and types of shear damage in jointed rock masses with different roughness during the shear process.

## Preparations for the shear test of jointed rock-like materials

Since natural rock mass are not homogeneous materials, natural rock mass or rock-like materials are often used for tests considering the non-homogeneous properties of rock mass. Due to the intricate structural features of rock mass in their natural state, it is challenging to find suitable natural rock mass in nature for testing. Therefore, this section adopts the research related to joint shear test by making rock-like materials. We prepared cylindrical specimens according to the proportion of rock-like materials, and conducted tri-axial compression and splitting tests to obtain the physical and mechanical parameters of the specimens, which provide the basis for the subsequent numerical simulation program of joint shear deformation and calibration of the material properties.

### Joint plane mold design and preparation

Due to the random nature of surface roughness in natural rock joints, it is necessary to quantify the degree of roughness on the joint surfaces to ensure the stability of experimental results. Barton et al.^44^ conducted numerous field tests and measurements on natural rock joints, extracting ten different levels of natural joint roughness. Based on this, they proposed the standard JRC (Joint Roughness Coefficient) curves, which provided a simple quantification and classification of surface roughness in natural rock joints. The standard JRC curves proposed by Barton are considered authoritative within the industry for quantifying joint roughness. In order to ensure the reproducibility and relevance of the research in this paper, ten commonly used standard JRC curves were selected as the research objects, and molds were designed and produced based on these curves.

**Figure 2:**
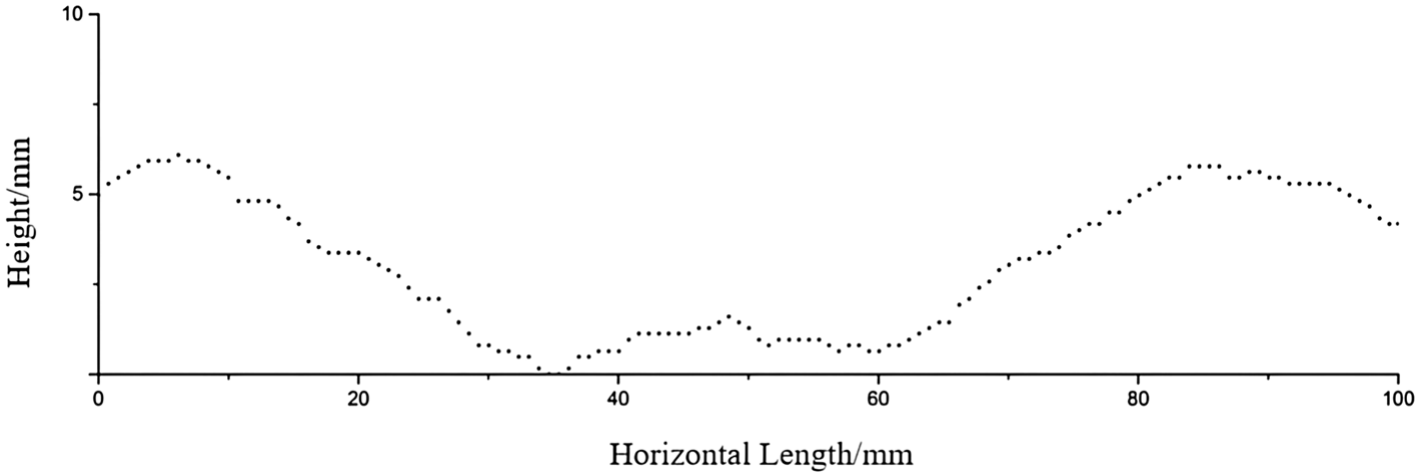
Discrete point extraction of standard JRC curves.

When creating molds for rock-like joint roughness specimens, the first step involves saving the JRC standard profile lines as image files, which are then imported into CAD software. Subsequently, the ten standard JRC continuous curves are evenly divided, discretizing the JRC continuous curves (see Fig. 2). Each contour line can be obtained with corresponding discrete points. Since the sampling interval of the standard JRC curves is 0.5 mm, the sampling interval of the contour lines should also be 0.5 mm or multiples of 0.5 mm.

**Figure 3:**
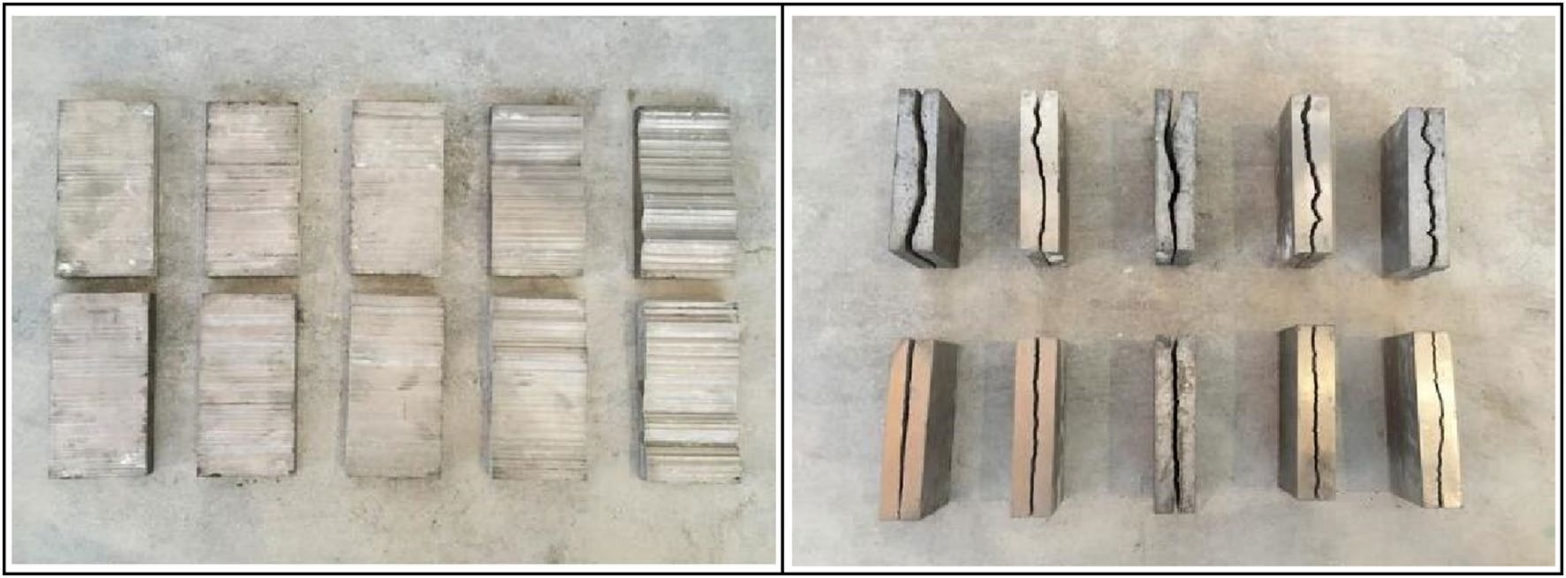
Standard JRC curve mold.

Using AutoCAD to divide the coordinates of the discrete points at equal distances and enlarge the coordinates at equal proportions, we cut the steel plate by a slow-feeding wire cutter from a steel plate mold manufacturer. The test piece molds with length, width, and height of 200 mm, 100 mm, and 50 mm were prepared, and the total number of test piece molds is ten, which corresponds to that of the standard JRC curves of the different ten contour lines, with an error of less than 0.05 mm. After the molds are prepared, half of the joint surface specimens based on the JRC curves are fabricated by inverting the molds. Using this half of the joint surface specimen as a mold, inverting the molds again allows for the production of the entire joint surface specimen. After completing the curing process for the joint surface specimens, they are ready for direct shear experiments (see Fig. 3).

### Determination of material ratios

In view of the similar material test of jointed rock-like material carried out in previous studies, we chose to conduct orthogonal test and selected uniaxial compressive strength and modulus of elasticity as the control indexes of material properties, and prepared rock-like similar materials according to natural rock mass. In this study, we did not consider the influence of filling material of the joint on the rock shear performance. Therefore, the natural conditions of rock mass joint plane and the mechanical properties of intact rock mass are consistent. However, in the preparation process of rock-like materials, the irregularity of the rough joint surface is relatively small, and the larger particles in the coarse aggregate are not easily accommodated in the fissures. This results in a significantly lower strength of the intact rock compared to the strength of the undamaged rock. Therefore, to ensure that the strength of the fracture surface roughness is consistent with that of the intact rock, this paper employs CA270 aluminous cement and corundum with smaller particle sizes.

Through the proportioning test of the jointed rock-like material, it is found that each material content ratio changes will change the mechanical parameters of the jointed rock-like material specimens, and a variety of materials interact with each other, but the overall mechanical properties still show a certain regularity: the proportion of CA270 aluminate cement content has a more obvious effect on the uniaxial compressive strength of molded jointed rock-like material specimens, and the uniaxial compressive strength can be improved by increasing the proportion of cement; the proportion of corundum content has a certain effect on the modulus of elasticity of the specimen, and the modulus of elasticity of molded rock-like material specimens can be reduced by increasing the content of corundum. In addition, a trace amount of water reducers was added in the proportion to improve the initial solidification speed of the material. Next, making the elastic modulus and uniaxial compressive strength as the control indexes, and according to the relevant rock parameters described in the Rock Mechanics Manual, we used the CA270 alumina cement, corundum, water reducer, and water for the proportioning to obtain CA270 cement, 0.5–1 mm corundum, 0–0.5 mm corundum, − 45 µ corundum, water reducer ADS3, water reducer ADW1. And the proportioning ratio is 1:2.4:1.4:1.2:0.018:0.042:0.4.

In addition, after the preparation of specimens is completed, the curing temperature and curing duration greatly influence the material strength of the jointed rock-like material specimens. Therefore, in the orthogonal test which determines the material proportion, we selected the fast-setting and high-strength alumina cement and prepared water reducers. We put the prepared specimens into 110 ℃ constant-temperature incubator for continuous curing for 24 h, which ensured that the specimens’ material strengths can meet the requirements and saved the test time to increase the efficiency.

### Equipment for testing

The equipment for testing involved in this study mainly includes tri-axial rock testing machine, rock shear percolation testing machine, curing box, drying box, shaking table and mixer, which were employed to determine of the proportion of rock-like materials, calibrate material properties, prepare specimens and conduct mechanical experiments, thus realizing efficient and stable recording and analysis of the mechanical shear properties of natural jointed rock mass in the lab.

#### Triaxial rock testing machine

**Figure 4:**
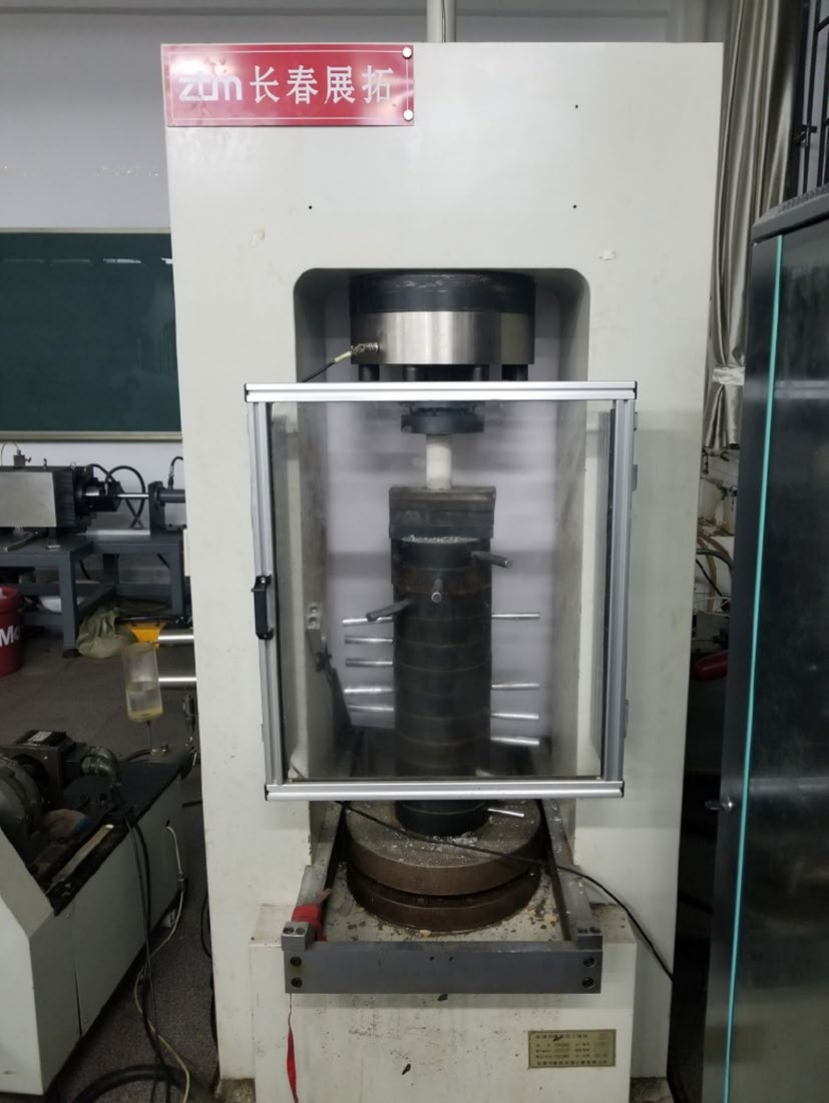
Triaxial testing machine.

The uniaxial compression tests of rock mass were completed in the laboratory of CCEA using the triaxial compression testing machine which consists of an axial pressurization system, a confining pressure stabilizing system, an axial pressure testing system, and a servo controller, as shown in Fig. 4). The tests were controlled by the servo controller, and the loads were exerted by the axial pressure system under a certain confining pressure applied by the confining pressure system in order to carry out the triaxial tests on the specimens of rock-like materials. Uniaxial compression can also be performed directly with no confining pressure applied.

(i) Confining pressure system

The confining pressure system is pressurized by hydraulic oil, and the hydraulic loading system is located under the shearing machine, and the hydraulic loading is carried out individually by jacks. The pressurization process is stable and reliable, and fully applicable to rock shear tests in lab.

- (ii) Axial pressurization system

Axial pressure system mainly consists of four parts: hydraulic pump, axial controller, pressurized oil pump, axial loading frame, etc. The tri-axial testing machine applies load through the hydraulic pump, and loads the cylindrical specimen vertically through the axial loading frame, and the experimental parameters such as the loading speed are controlled through the servo control system, which is stable and controllable.

- (iii) Axial pressure test system

The axial pressure test system was employed to test the pressure changes reliably, and to record the testing data in the testing process. It is made of metals with good elasticity. The deformation of the pressure was measured by the displacement sensors, and then converted to real-time axial pressure value.

- (iv) Servo stabilizer

During the uniaxial compression test, all testing operations were carried out through the servo controller on the testing machine loading system for accurate control. The EDC servo regulator, connected to the control computer, is utilized for controlling and regulating the loading rate, loading displacement target value, and loading load target value. This enables the computer to achieve the goal of measuring and obtaining the specimen changes after applying different loads, facilitating the investigation of the uni-axial and tri-axial mechanical properties of the column specimens.

#### Rock shear seepage testing machine

**Figure 5:**
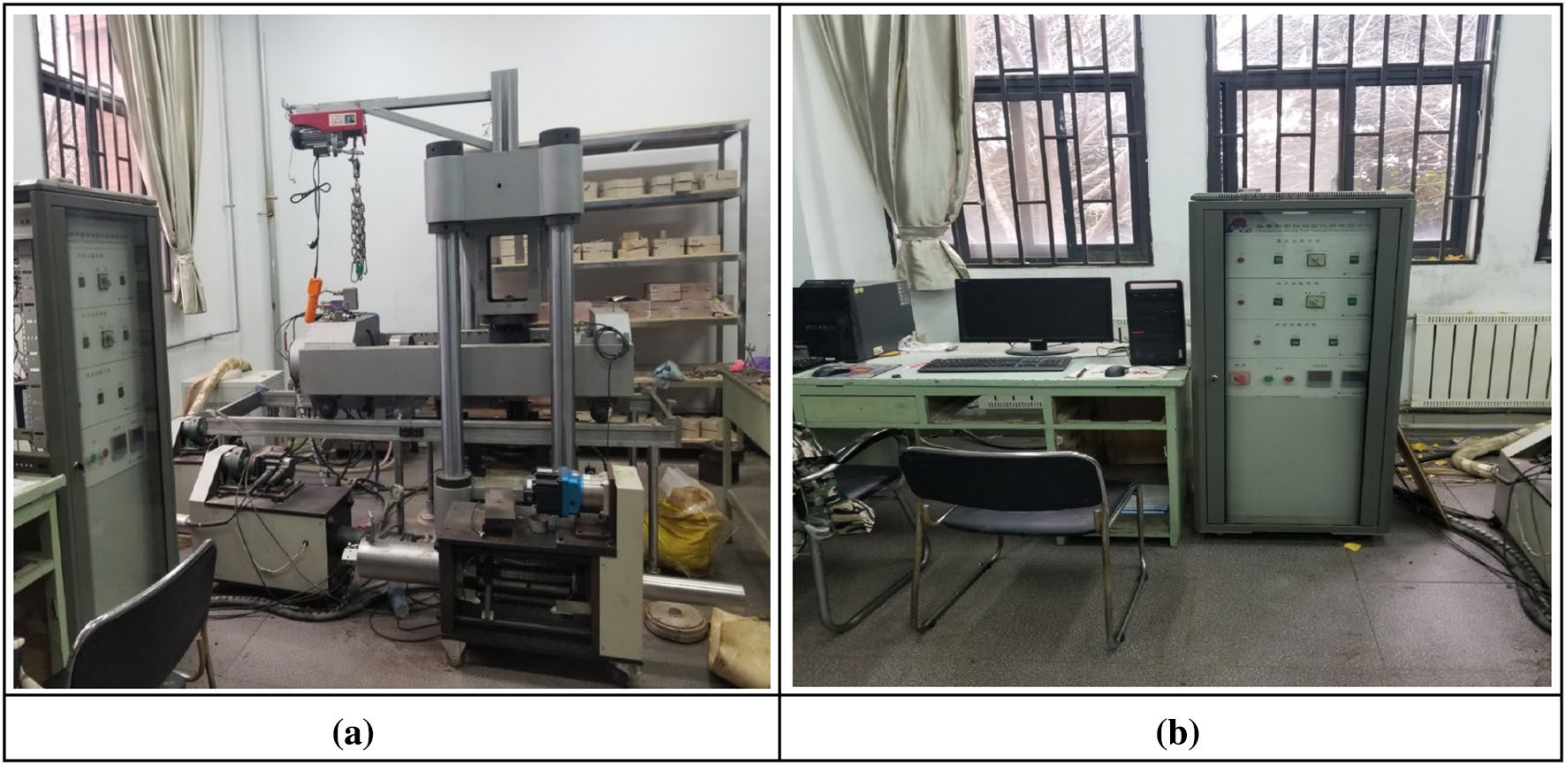
Shear seepage testing machine. (**a**) Straight shear testing machine. (**b**) Servo test system.

This test was completed in laboratory 120 at CCEA using JAW-600 rock shear seepage testing machine (see Fig. 5). The main structure of the system includes a four-column loading frame, under which the cylinder is located. The frame is beautifully structured with good rigidity. The test machine uses a DOLI fully-digital servo pressure control system imported from Germany, which can realize safe, efficient and reliable pressure control and simple operations. The computer system performs reliably and can accurately read and set the whole process of shear tests. The main components of the axial pressure system, confining pressure system, servo control system and computer system are as follows.

- (i) Axial pressure systems

The loading frame, servo oil source, etc. constitute the axial pressure system of the testing machine.When this system is running, the servo oil source provides oil pressure, and the loading frame exerts the pressure, and the pressure oil is under the control of the servo valve for the application of normal load. With the increase of the pressure oil, the piston of the loading frame rises slowly, and the specimen and the frame gradually come into contact with each other, and then axial load is applied after the contact.

- (ii) Confining pressure system

Confining pressure loading system, pressure chamber and confining pressure controller, etc. constitute the confining pressure system of the testing machine. The confining pressure loading is controlled by servo motors; pressure chamber is easy and inexpensive for both installing and uninstalling, and is able to accurately measure the deformation and displacement of the tested specimen in shear; confining pressure controller adopts the imported DOLI servo controller, which features reliable and accurate control and makes the confining pressure system able to complete the tests efficiently and accurately.

- (iii) Servo control system

This system mainly consists of axial control system, hydraulic control system and operating system, etc., which can control and monitor the testing process. With the usage of the EDC servo controller, all kinds of data sensed are amplified and processed, and the data measured by the sensor are collected and displayed in the form of data and curve through the computer system. Besides, it also uses the servo valve to improve the adjustment of the unsatisfying testing process, so that the tests can accurately and stably simulate the real rock stress conditions. The sensors mainly include the load and displacement sensors, which can accurately record the displacement, damage and force of rock-like materials.

- (iv) Computer system

The whole testing process is mainly controlled, recorded and adjusted by this computer system. Through the interaction between the testing equipment and the EDC controller, the mechanical properties of the rock-like materials are monitored and recorded in real time during the tests, and the curves are plotted as required. At the end of the test, it can extract, summarize and analyze the data.

#### Other testing equipment

**Figure 6:**
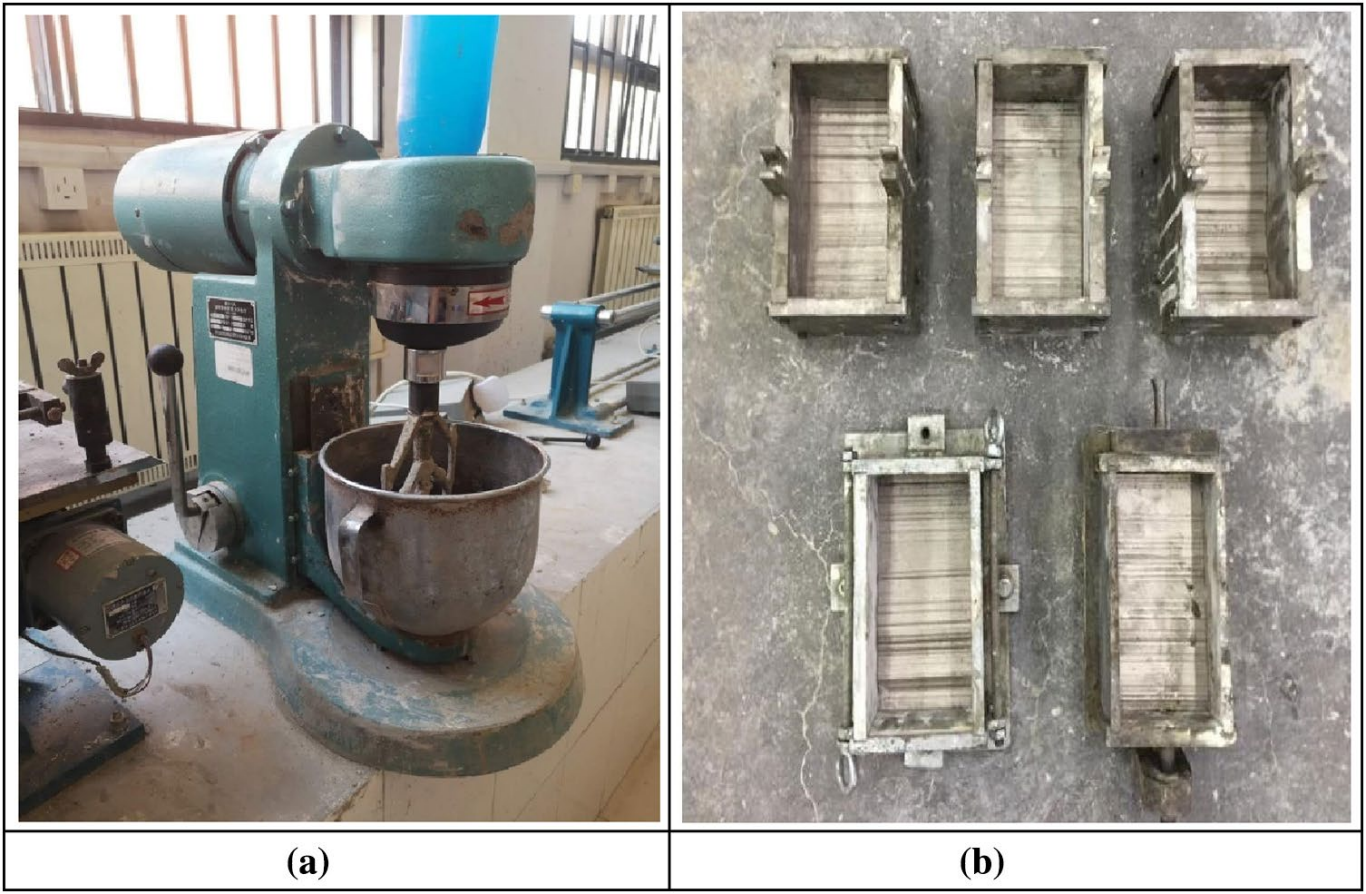
Other test equipment. (**a**) Planetary mixer. (**b**) Joint plane steel mold.

During the tests, some other testing equipment was used for the preparation and curing of the rock-like material specimens (see Fig. 6). In the preparation of the specimens, the cement material was mixed using the JJ-5 cement mixer in the test hall. The volume of the JJ-5 mixer is five liters and the width of the mixing fan is 135 mm. While rotating on its own, the mixing fan is also rotating along the mixer seat. After the mixing, the vibrating table is used for vibratory compaction, which vibrates the mixed specimens with constant amplitude and vibration frequency, and then the specimens are dried and maintained by the drying box and the maintenance box. Prepare cylindrical specimen molds with a height-to-diameter ratio of 2:1 and a height of 100 mm to determine the material properties of the rock-like material through uniaxial compression tests. Prepare ball bearing steel plate and thin steel plate. The ball steel plate can ensure that the end of the specimens can move stably when subjected to shear, and the thin steel plate can ensure that the dense end load of the ball bearing steel plate will not crush the specimen.

### Preparation and curing of jointed rock-like material specimens

After determining the material proportion of rock-like materials and preparing the joint mold as well as the specimen mold, specimens with similar material properties as those of natural rock mass were prepared. Under the action of equipment as close as possible to the natural geological conditions, the joint plane damage tests were conducted, which restored the stress reality of the natural jointed rock mass in the natural conditions and provides reliable test data for joint studies.

#### Preparation of jointed rock-like material specimens

According to the pre-determined rock-like material ratio, various materials of the specimen are weighed using high-precision balance, and each rock-like material specimen is individually weighed. After weighing, all kinds of materials were poured into the metal disk and stirred evenly, then poured into the mixing bucket. Turn on the mixer, and add water according to the proportion. After five minutes of mixing, the well-mixed materials were poured into the specimen molds in which the joint molds had been placed. Put the mold on a vibration table for being vibrated to compact the rock-like material and allow the air bubbles generated by the mixing of the material to escape. After ten minutes of vibration, the specimen molds were removed and the specimens were placed on a horizontal surface for 24 h. After resting, put the specimen into the constant-temperature curing box for a period of time.

#### Curing of jointed rock-like material specimens

This test used CA270 Aluminate Cement, which features high strength and quick drying. The strength of the rock-like material specimens reached a certain degree after 24 h of resting. Since aluminum cement is a hydrophilic material, it is very easy to absorb moisture in the air, which will cause chemical reaction and failure. However, the relative air humidity in Qingdao where CCEA is located is relatively high (averaging 70%). During the preparation and curing of the specimens, the high humidity can greatly affect the final strength of the specimens.

**Figure 7:**
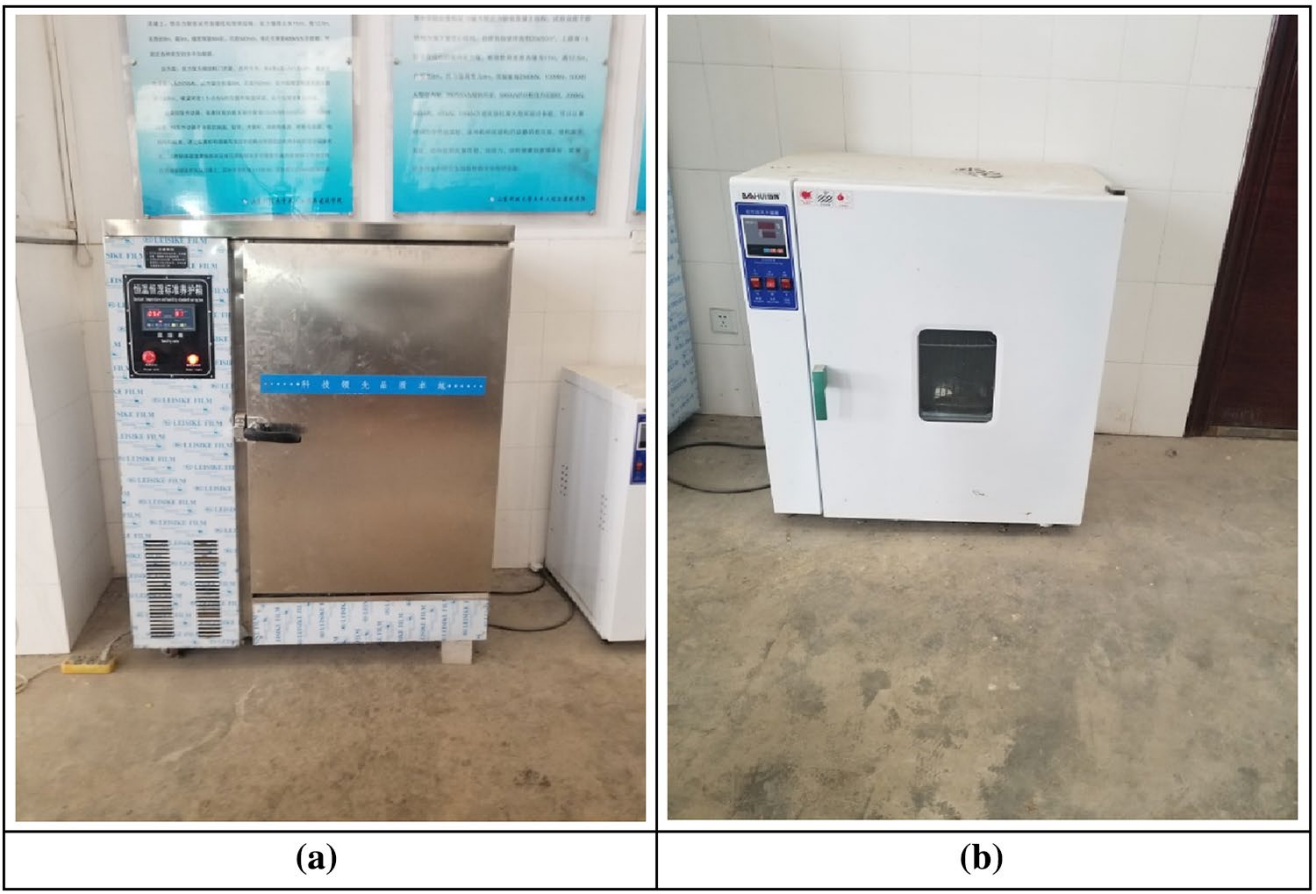
Test specimen curing equipment. (**a**) Constant-temperature curing box. (**b**) Drying box.

Therefore, in the process of specimen preparation and curing, attention should be paid to keeping the material dry to avoid material failure or strength reduction due to excessive moisture in the environment. In the process of specimen preparation, the test materials were first poured into the metal disk, and then put into the drying box to maintain a high temperature of 110 °C for drying for 24 h before the specimen preparation. After the preparation, the rock-like material specimens were put into the curing box for curing under the conditions of constant temperature and humidity for 28 days. And after 28 days, they were taken out for related tests (see Fig. 7).

### Determination of rock mechanical parameters of jointed rock-like materials

We conducted the uniaxial compression test on the prepared cylindrical specimens of the rock-like materials to obtain their uniaxial compressive strength, modulus of elasticity and Poisson’s ratio, and then conducted triaxial compression test to obtain the internal friction angle and adhesion of the rock-like materials. Finally, we obtained the basic friction angle of the structural planes of the rock-like materials by the straight shear test of the flat joints. We obtained various required material properties by performing compression and straight shear tests on the rock-like materials. In the uniaxial compression tests, displacement control was used and the pre-peak and post-peak loading rates were taken as 0.08 mm per second and 0.1 mm per second, respectively, with a height-to-diameter ratio of 2:1 and a height of 100 mm. Straight shear tests were carried out on flat joints under different normal loads with the loading rate set at 0.6 mm per minute, and tri-axial compression tests were carried out on the rock-like material specimens by applying different confining pressures with the loading rate taken as 0.01 mm per second. It can be obtained from the tests that the main damage form of the cylindrical specimens is split tensile damage. The measured mechanical properties of the corresponding rock-like materials are shown in Table 1.

**Table 1:** Mechanical properties of specimens.

Parameters	Measurements
Compressive strength (MPa)	102.35
Young’s modulus (GPa)	32.05
Density (kg/m^3^)	2300
Internal friction angle (°)	35.88
Poisson’s ratio	0.117
Internal cohesion (MPa)	25.43

## Shear test on jointed rock mass with different shear conditions

In nature, as a result of geological tectonic movements, intact natural rock masses are split into blocks, creating rocks containing joints which can greatly affect the shear strength of the rock masses. In this section, we conducted the shear tests on specimens of rock-like materials to simulate the staggered slip movement of closed joints of natural rock mass under natural conditions. In this experiment, five sets of roughness joint specimens were prepared, including flat joint and JRC standard joint curves 2, 6, 8, and 10. The specimens prepared according to the JRC standard joint curves 2, 6, 8, and 10 were respectively labeled as S1, S2, S3, and S4. We tested joint planes with different roughness to record and investigate the shear performance of jointed rock mass with different roughness. We adopted the displacement-controlled method to shear the rock-like material specimens with different rates to record and investigate the shear performance of jointed rock mass with different shear rates. Also, we applied different confining pressures to the rock-like materials to record and investigate the shear performance of the jointed rock mass under different confining pressures.

### Analysis of shear displacement characteristics and damage patterns of jointed rock-like materials

**Figure 8:**
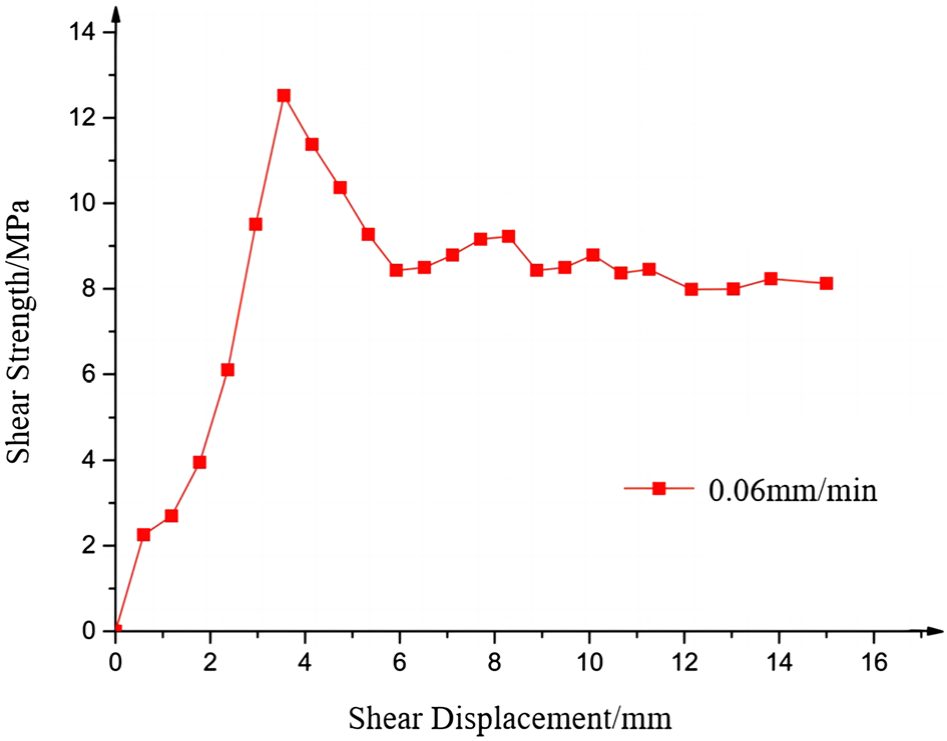
Shear strength-displacement curve of joint specimens.

During shearing, axial stresses were applied to the rock-like material specimens, and after the normal stresses were stabilized to the target value, the specimens were subjected to straight shear tests at a fixed rate. The specimen labeled as S1 was subjected to shear testing at a shear rate of 0.06 mm/min under an axial stress of 10 MPa, resulting in the shear stress-displacement curve of rock-like joint specimens (see Fig. 8). The whole shear process roughly includes four stages: compaction stage, elastic deformation stage, plastic deformation stage, and residual shear strength stage. During the initial loading on the rock-like materials, the relative displacement of the closed joints occurs under the action of transverse shear displacement. Since the closed joints are not perfectly adherent to each other, the joint planes are not in complete contact with each other during the initial stage of shearing. In the shear stress-displacement curve, this process is shown as a slightly upward convex curve, and after the shear reaches the friction peak, there is a small displacement. The stress change during this process is relatively small due to the voids in the joint planes until the joints are completely compressed tightly to each other. When the joints are completely closed, only the joints undulating surface bears the shear displacement, and it marks the entering into the elastic shear stage. With the increasing shear load, the rock-like material specimens under the joint action of compression and shear show elastic deformation. And in the elastic deformation stage, the shear stress-displacement curve becomes close to a straight line going up, and in a relatively short period, it reaches the peak shear value. After elastic deformation, the shear displacement of the specimens increases continuously, and the specimens gradually enter the plastic stage. And when the rock-like material specimens reach the yield point, the specimens experience plastic deformation. However, since the properties of rock-like materials are similar to those of natural rocks, the process of plastic deformation is very short and brittle damage occurs rapidly in the specimens. The shear strength of the jointed rock mass, after reaching its peak value, decreases sharply with the appearance of brittle damage, and in this stage the curve is characterized by a downward convex shape. This is due to the fact that the shear stress decreases as the joint plane experiences fracture microscopically due to brittle damage during the plastic deformation stage. As the shear displacement continues to increase, the joint plane exhibits strain softening phenomenon, and the shear stress turns stable, with the magnitude of change gradually becoming smaller. As the undulating surface is completely destroyed, with the advancement of shear, the joint planes of the specimens enter the residual shear strength stage, and the curve basically remains level with much smaller changes.

**Figure 9:**
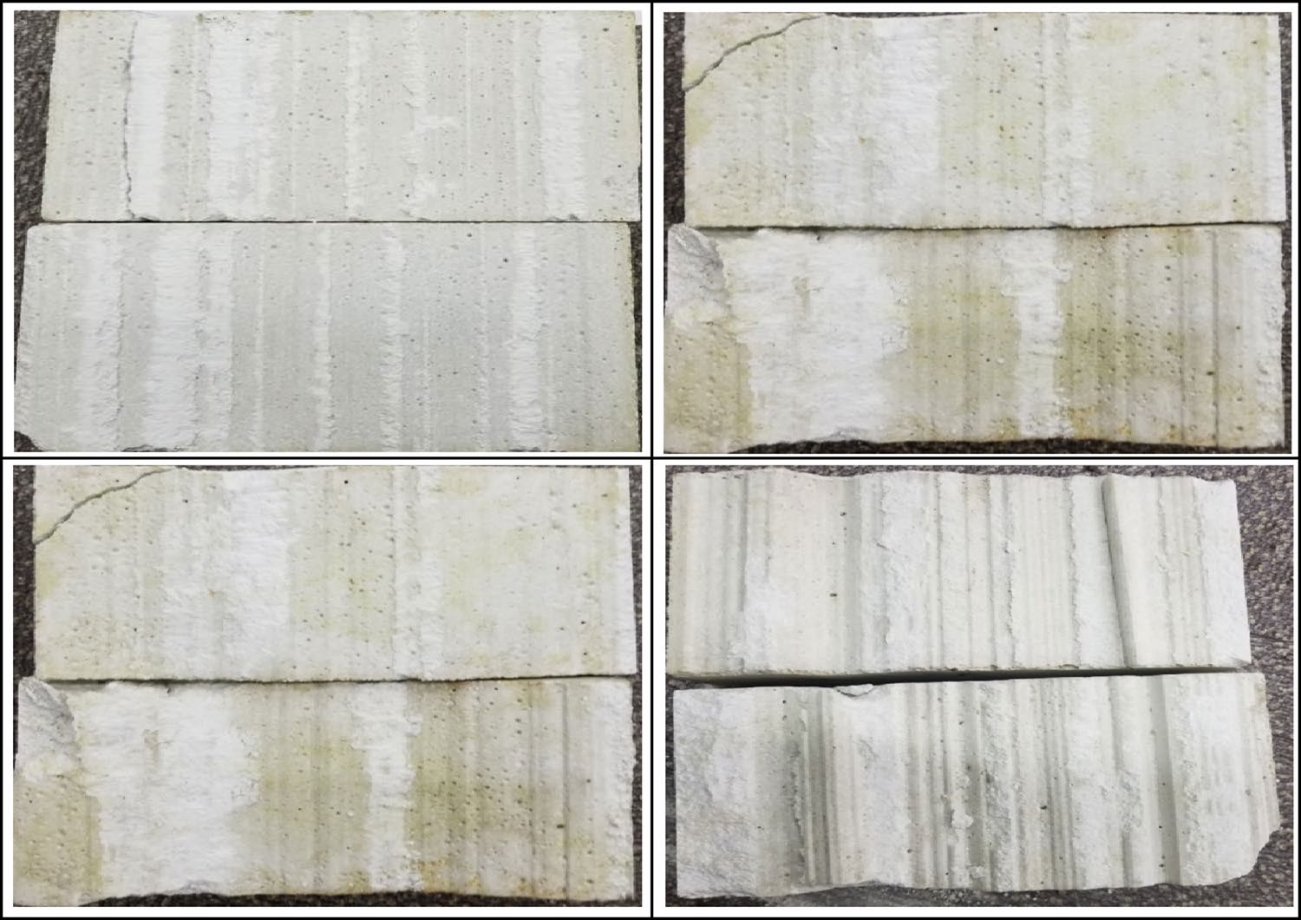
Surface damage of jointed rock mass.

**Figure 10:**
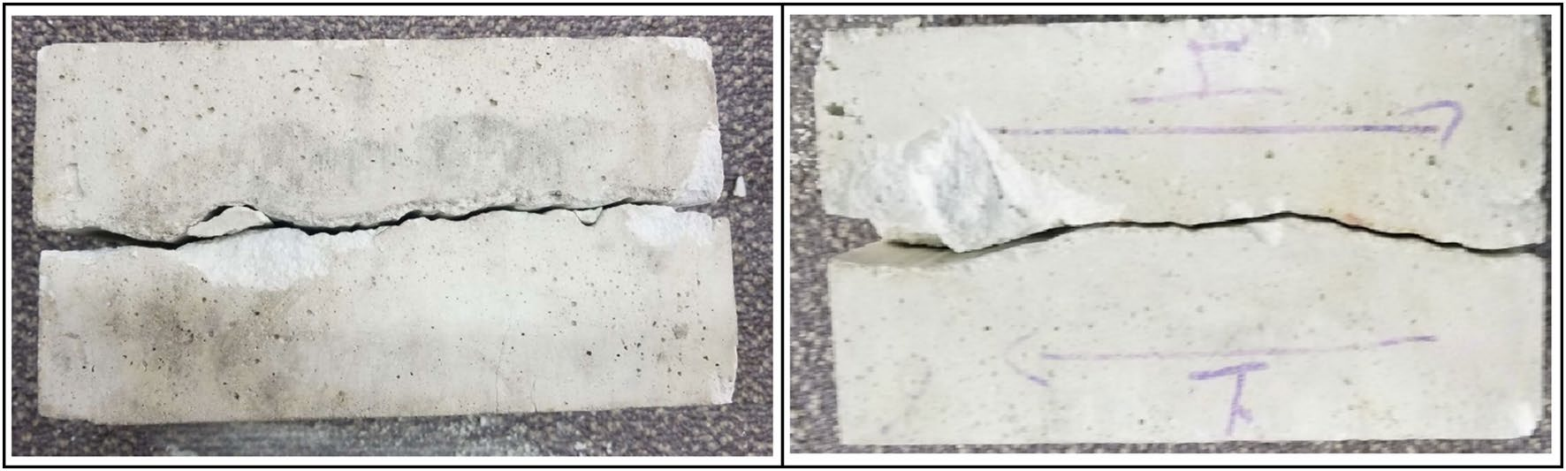
Side damage of jointed rock mass.

By observing the surface damage (see Fig. 9) and the side damage of the jointed rock mass specimens (see Fig. 10), it can be obtained that the shear damage of the rock-like materials is mainly abrasion and fracture damage. The fracture damage occurs in the part of the joint plane with larger degree of undulation, and abrasion damage occurs in the part of the joint plane with smaller degree of undulation. The whole specimens exhibit crack damage from the joint plane to both sides and spreads and propagates. The cracks are distributed uniformly on the joint plane, and the cracks are more concentrated on the protrusions on the joint plane. During the tests, no obvious plastic damage was observed, and the rock-like material specimens mainly experienced brittle damage.

### Effect of joint roughness on the shear performance of rock-like specimens

**Figure 11:**
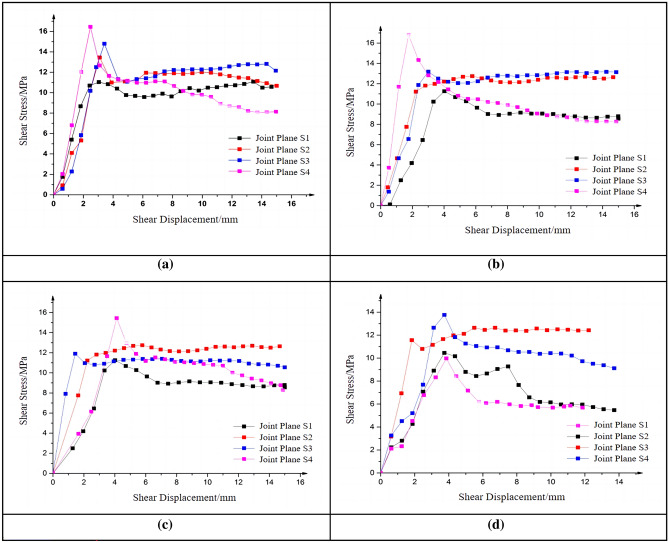
Shear stress-displacement curves of specimens with different roughness. (**a**) Shear rate of 0.06 mm/min. (**b**) Shear rate of 0.6 mm/min. (**c**) Shear rate of 6 mm/min. (**d**) Shear rate of 12 mm/min.

The influence of roughness on the shear performance of joint plane was investigated by conducting straight shear tests on joint planes with different roughness and analyzing the test results. Besides, through the straight shear test under a variety of shear rates, summarize the effect of different roughness on the shear stress law. The shear stress-displacement curves of joint planes with four different roughnesses under the same shear rate and confining pressure are listed (see Fig. 11).

The specimens labeled as S1, S2, S3, and S4 were subjected to direct shear tests under shear rates of 0.06 mm/min, 0.6 mm/min, 6 mm/min, and 12 mm/min, respectively. The shear stress variations under different roughness conditions will be compared. As shown in Fig. 11, reveals that under the test conditions of the same shear rate and confining pressure, the roughness of the joint plane has an obvious influence on the shear strength of the specimens. The higher the roughness of the joint plane, the higher the shear strength of the joint plane.

The shear stress-displacement curves show that joint planes with greater undulations have higher peaks and require smaller shear displacements to reach the peak. It can also be found from the curve that with the steady increase of shear displacement during the shear process, some of the shear stresses of the specimens in the elastic stage do not increase in a nearly straight line as expected, but exhibit some steep changes. And before and after the steep changes, the shear stresses with the shear displacement increase at nearly the same slope. The reason for the steep changes may be that during the shear tests, although the shear specimens are in the elastic stage, local parts of the specimens still experience some small damages due to large roughness of the joint plane and some inevitable operation errors in the specimen preparation process, resulting in the reduction of specimen shear strength. This can be also observed in the test process.

**Figure 12:**
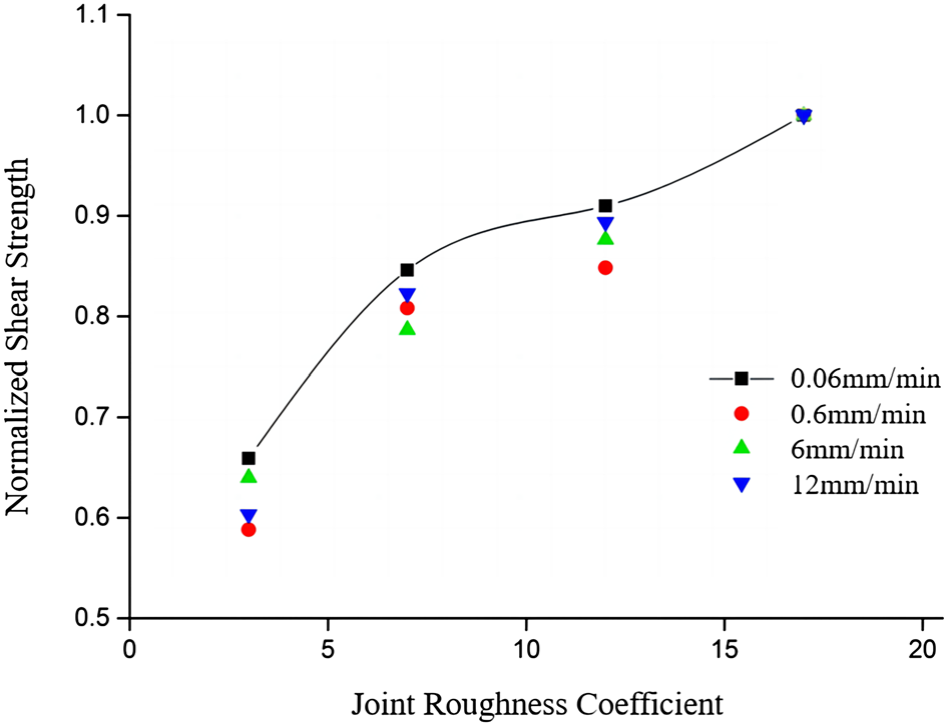
Normalized shear strength changes with different roughness.

Table 2 and Fig. 12 show the normalized roughness coefficients, and the trend of shear strength growth can be observed more intuitively through Fig. 12. From the normalized coefficients, it can be found that with smaller roughness, the shear strength grows faster, while the roughness becomes larger, the shear strength grows relatively slower. This may be attributed to the increase in roughness, which leads to a higher density of small protrusions on the joint surface, and consequently, fails to effectively enhance the peak shear strength of the specimen.

**Table 2:** Normalized shear strength with different roughness.

Shear rate (mm/min)	Normalized peak shear strength			
	S1	S2	S3	S4
0.06	0.659	0.846	0.909	1
0.6	0.588	0.808	0.848	1
6	0.639	0.786	0.876	1
12	0.603	0.822	0.893	1

The shear strength of rock mass corresponding to different joint plane roughness is very different, comparing the peak strength of flat joints with the shear strength of joints with different roughness, the increase is as follows Table 3.

**Table 3:** Variation of peak shear strength of different roughness.

	Peak shear strength/MPa	Range of change	Residual shear strength/MPa	Range of change
Flat joint	7.75	0	5.85	0
S1	11.065	42.77%	10.4	77.78%
S2	13.667	76.35%	10.68	82.56%
S3	14.3698	85.42%	12.3654	111%
S4	16.572	114%	7.82	33.68%

Comparison of the changes in peak shear strength and residual strength between flat joints and joint planes with different roughness reveals that the roughness enhances the shear strength of flat joints significantly. However, it is also found that the residual shear strength of joint plane No. S4 shows a steep drop, which, through analysis, may be due to the larger roughness that causes serious damage to the jointed rock mass, and more small protrusions are destroyed. Since the later destruction of the jointed rock mass with larger roughness is more serious, more attention should be paid to the support cleaning and reinforcement after the destruction of the rough rock masses in the actual engineering construction process.

### Effect of shear rate of joint plane on the shear performance of rock-like specimens

**Figure 13:**
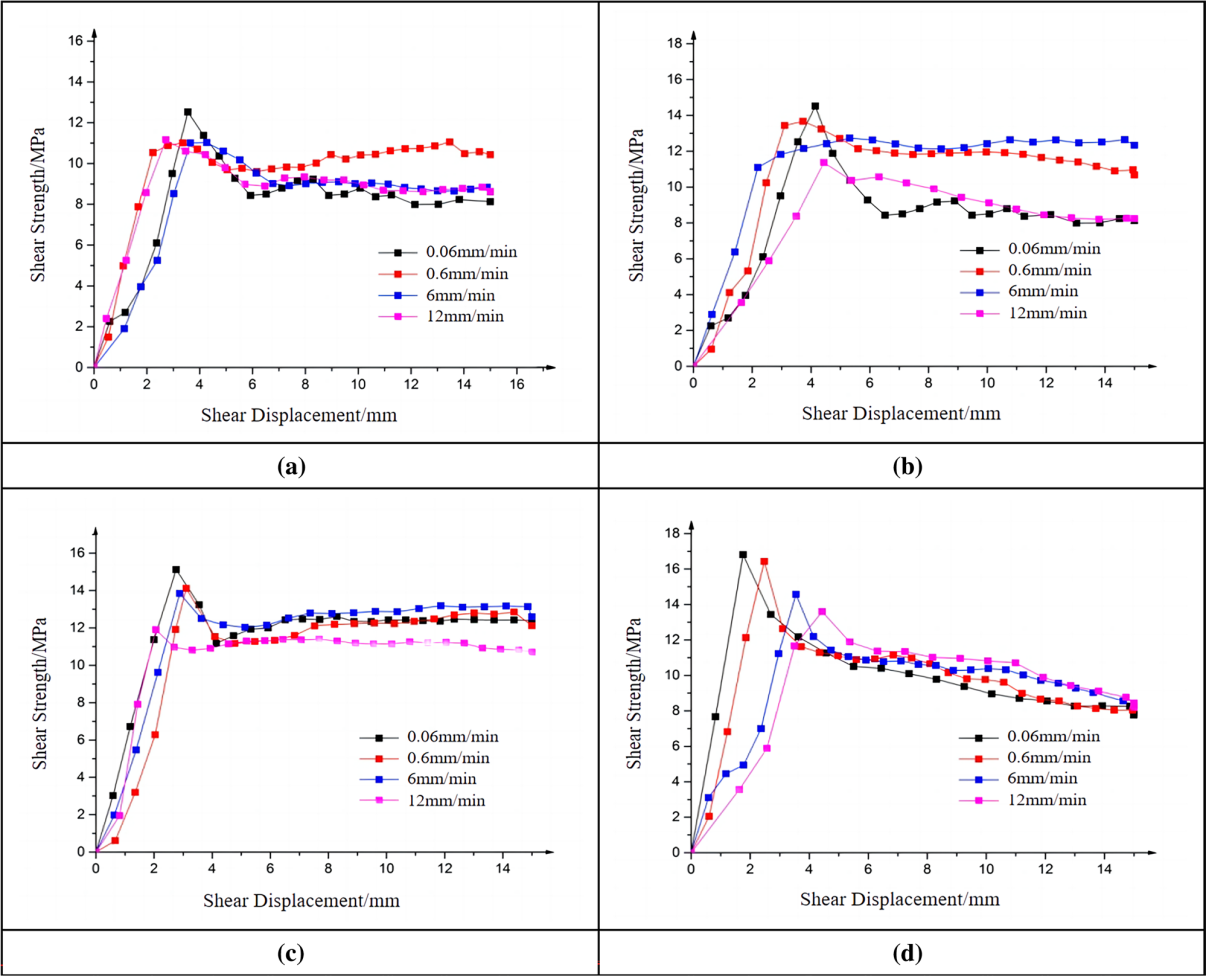
Shear strength-displacement curves of specimens with different shear rates. (**a**) Joint plane No. S1. (**b**) Joint plane No. S2. (**c**) Joint plane No. S3. (**d**) Joint plane No. S4.

Figure 13 below shows the shear strength characteristics of specimens with the same roughness and normal load at different shear rates of 0.06 mm/min, 0.6 mm/min, 6 mm/min and 12 mm/min. And direct shear tests were conducted at these four shear rates on the joint planes with different roughness.

The rock-like material specimens were sheared at the rates of 0.06 mm/min, 0.6 mm/min, 6 mm/min, and 12 mm/min to explore the effects of different shear rates on the shear performance of jointed rock mass. The results are shown in Fig. 13. The investigation revealed a gradual decrease in peak shear strength with an increase in shear rate. We obtained the normalized peak shear strength values of the four groups of specimens to research the joint specimen shear rate and peak change trend law.

**Figure 14:**
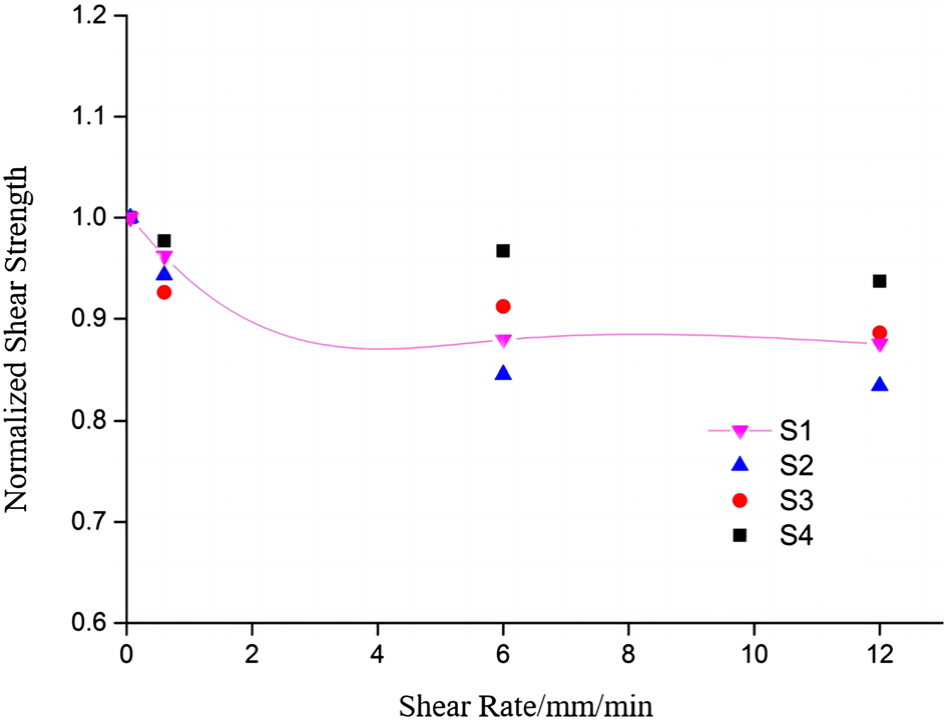
Normalized shear strength under different shear rates.

From Table 4 and Fig. 14, it can be observed that normalized peak shear strength significantly declines, and the decline rate of 0.06–6 mm/min section is higher, the decline rate starts decreasing in the 6–12 mm/min section and then the peak shear strength begins to stabilize. The results indicate that at relatively slow shear rates, the impact of shear rate on the peak value is more pronounced. However, once the shear rate surpasses a certain threshold, the influence of shear rate on the peak shear strength becomes less evident.

**Table 4:** Normalized shear strength at different shear.

	Normalized peak shear strength			
	0.06 mm/min	0.6 mm/min	6 mm/min	12 mm/min
S1	1.000	0.957	0.879	0.872
S2	1.000	0.948	0.848	0.835
S3	1.000	0.925	0.916	0.896
S4	1.000	0.979	0.968	0.957

### Effect of confining pressure on the shear performance of rock-like specimens

**Figure 15:**
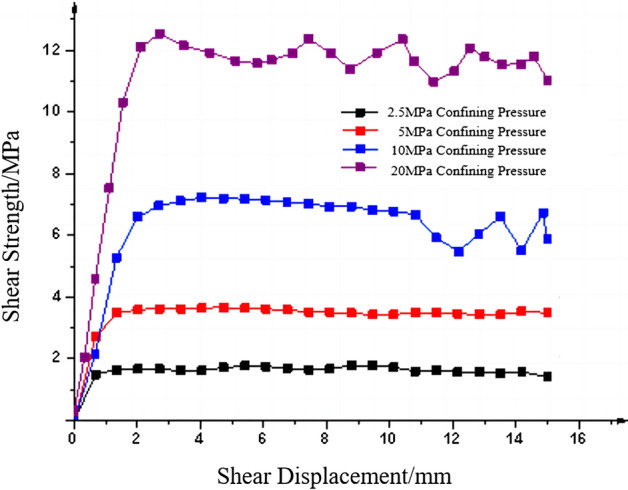
Shear strength-displacement curve of specimens under different confining pressures.

Figure 15 illustrates the shear strength-displacement curves of the specimens at a shear rate of 0.6 mm/min under varying confining pressures. The normal loads of 50 kN, 100 kN, 200 kN and 4000 kN were applied to the four flat joints, respectively, to study the variation law of the shear stress and the peak value of the specimens under different confining pressures.

The confining pressures of 2.5 MPa, 5 MPa, 10 MPa and 20 MPa were exerted on the specimens, respectively, for straight shear test to study the effect of different confining pressures on the shear performance of the jointed rock mass. The experimental results, as shown in Fig. 15, reveal that the shear strength is positively proportional to the confining pressure. The shear strength-displacement curve is basically a relatively smooth curve, but under the 20 MPa pressure, after the shear strength reaches the peak, the specimens exhibit more obvious failure fluctuations, which are manifested as up and down of the curve. This may be due to the relative misalignment of the joint planes under the shear displacement movement after a certain degree of confining pressure is reached. Under the 20 MPa confining pressure, the shear stress directly destroys the undulating surface since the jointed rock mass cannot be sheared along with the undulating surface. As shear displacement increases, the raised part of the undulating surface is directly sheared, and the shear strength shows a steep change. After shearing, the jointed rock mass specimens continue to remain pressure-tight under high confining pressure. With the increasing shear displacement, the undulating surface is continuously sheared, which is manifested as a steep change up and down in the curve.

**Figure 16:**
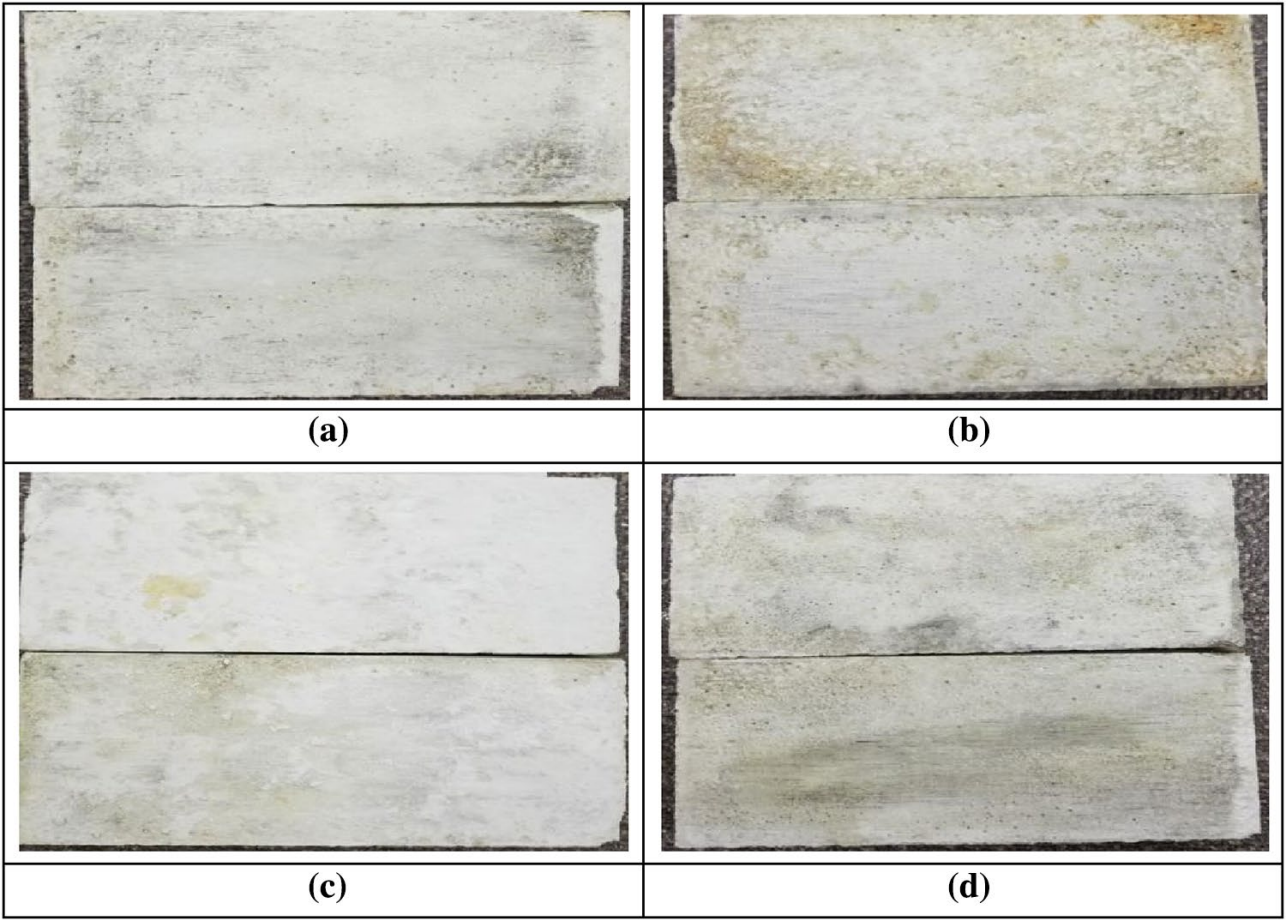
Joint plane with different confining pressures. (**a**) 2.5 MPa Confining pressure. (**b**) 5 MPa Confining pressure. (**c**) 10 MPa Confining pressure. (**d**) 20 MPa Confining pressure.

Figure 16 shows the damage of flat joint planes under different confining pressures, from which it can be seen that the flat joints is mainly subjected to abrasion damage, and there is no brittle fracture damage. It can also be observed in the shear strength-displacement curve in Fig. 15 that after the jointed rock mass specimens reach the peak shear strength, the shear strength decreases insignificantly. The residual shear strength of the jointed rock mass specimens is basically equal to the peak shear strength.

It can be concluded that local weak rock layers have a relatively small impact on shear strength in natural geological conditions. However, if local undulating surface strength is relatively weak, the shear displacement of the jointed rock mass will become larger to reach the complete brittle destabilization; in the process of shear, the distribution of the cracks is more concentrated on the undulating surface due to large undulation, especially on the protrusions on the joint plane.

## Analysis of mesoscopic displacements of jointed rock mass under direct shear

### Parameter selection and validation of numerical models

Complex geological structure and long years of crustal movement have formed the complex internal structure of natural rock masses, and led to the uncertainty of the internal mechanical and geometric properties of the rock masses, which makes it difficult to obtain the mesoscopic damage conditions inside the rock masses from lab tests. In order to simulate the development and fracture of jointed rock mass under shear, on the ABAQUS platform, we took the straight shear process of the rock mass with different roughness as the research object, and adopted the method of globally embedding the zero-thickness cohesive unit to establish the compression and shear test model^18,19^.

**Figure 17:**
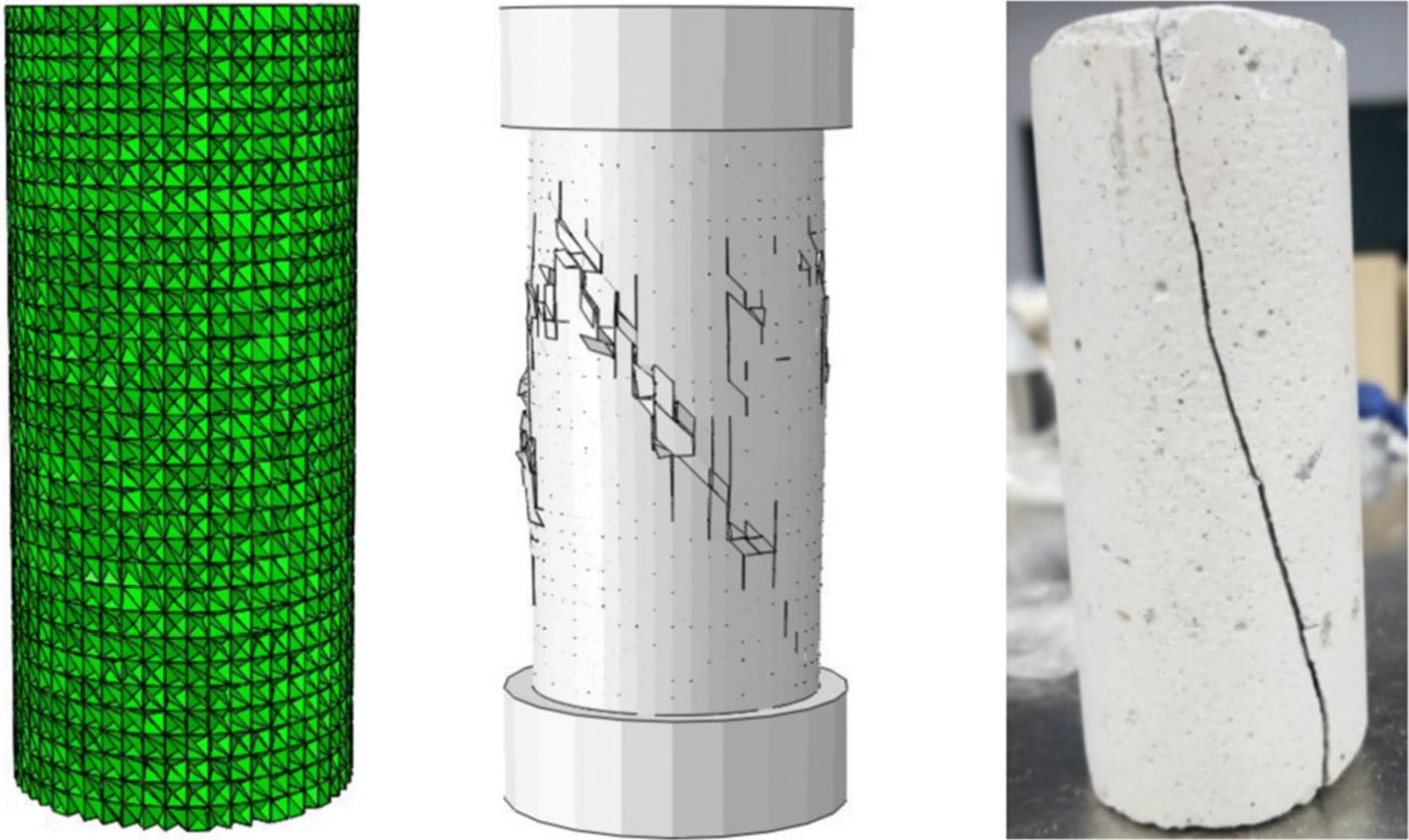
Numerical model of uniaxial compression and its failure.

As shown in Fig. 17, the constructed model should be equivalent to laboratory experiments. In order to obtain the shear mechanical parameters in the constructed model, a uniaxial compression test model with a height of 100 mm and a diameter of 50 mm was established. The parameters of rock specimens in Table 1 were incorporated into the numerical model, with the bottom boundary fixed, and a force equivalent to that of laboratory experiments was applied to the top to achieve a displacement rate of 10^−1^ mm/s. As illustrated in Fig. 17, all failure modes exhibited inclined fractures, with the solid lines representing the fracture zones, and the angles and shapes of the cracks closely resembling the experimental results, validating the scientific validity of the constructed numerical model. Through the method of “trial and error”^63^ —continuously fine-tuning the parameters of the numerical model to match the fracture of laboratory specimens. At this point, the finely tuned numerical values can be used for parameter calibration in shear simulation, and the calibrated material parameters of the numerical model can be obtained, as shown in Table 5.

**Table 5:** Numerical model material parameter calibration.

Materials	Parameters	Value
Rock-like material solid element	Density (kg/m^3^)	7500
	Young’s modulus (GPa)	200
Rock-like material cohesive element	Density (kg/m^3^)	2300
	Rigidity (N/m^3^)	19e12
	Fracture energy	0.188

### Establishment and verification of shear model

**Figure 18:**
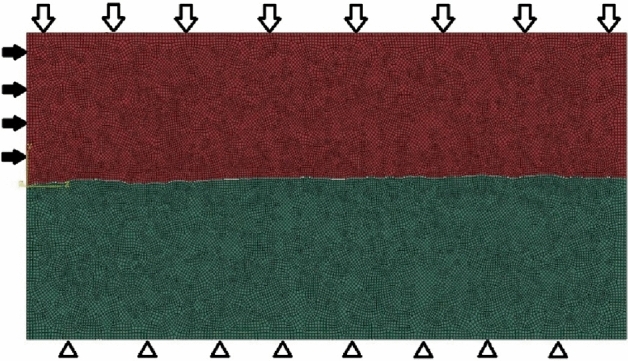
Shear mode of jointed rock mass.

**Figure 19:**
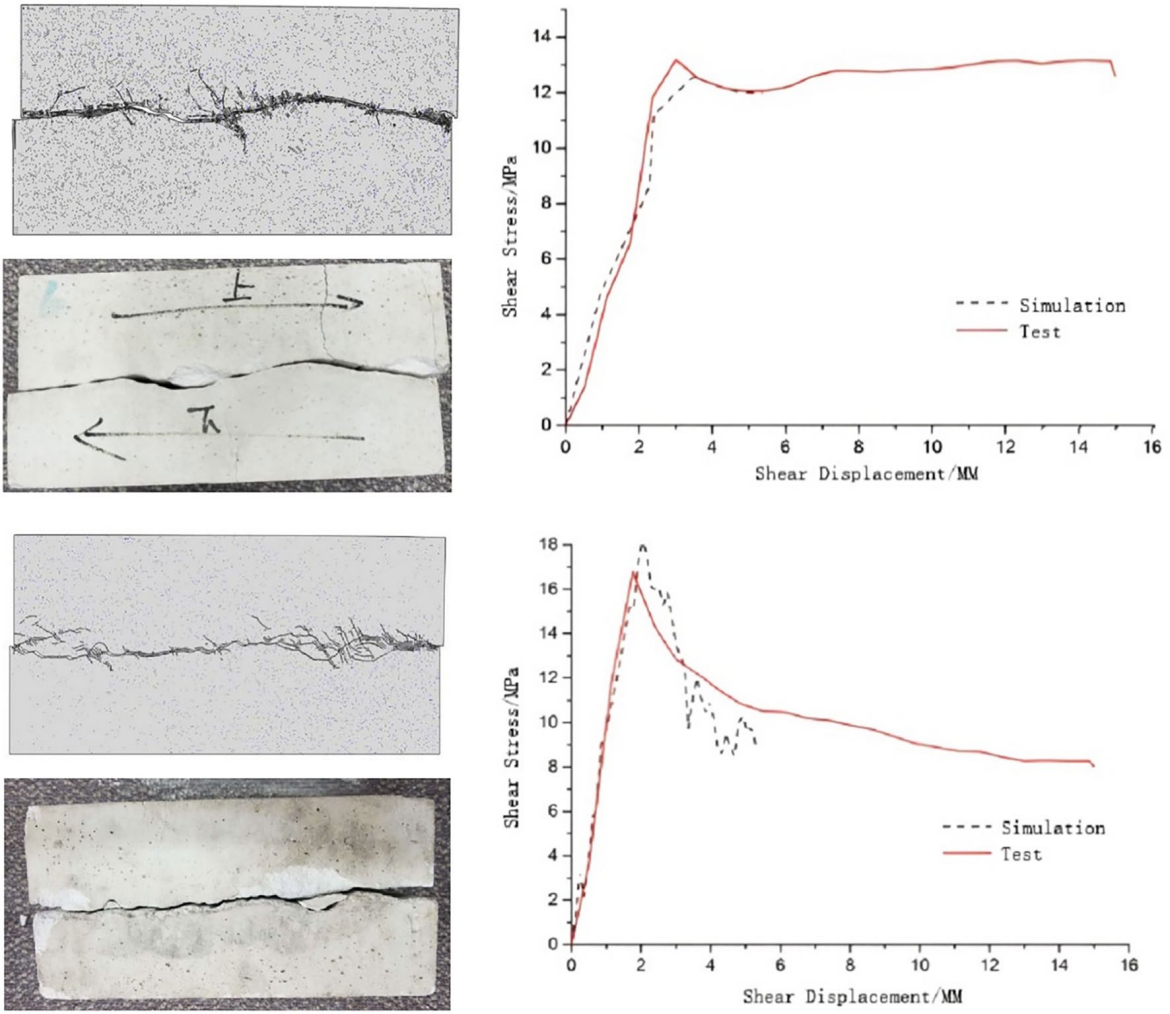
Comparison of test results and numerical simulation results (S3 and S4).

For investigating the internal mesoscopic displacement of jointed rock mass when subjected to direct shear, and analyzing the damage area and damage degree, we established a numerical shear model by embedding a zero-thickness cohesive unit using the ABAQUS traction separation model, which was of the same size as the specimen. The model’s upper surface bears a uniform downward load of 10 MPa, with the lower surface globally constrained. A consistent horizontal displacement is applied from left to right on the upper half of the model. The model is illustrated in Fig. 18. Comparisons were made between the simulation results from the numerical model and the test results, so as to ensure the scientificity and accuracy of the straight shear test calculation. As shown in Fig. 19, the shear stress-displacement curves and damage patterns obtained from the established model are quite similar to those obtained from the lab tests.

### Analysis of mesoscopic displacements of jointed rock masses under direct shear

We explore the influence of the joint plane on specimen deformation by examining the variations in internal displacement differences along the joint plane under direct shear loading. Subsequently, we analyze the regions of failure and assess the degree of damage. As found in the previous lab tests, the cracks are mainly concentrated in the protruding part of the joint plane. Considering that the internal displacement difference of the rougher joint plane may be more obvious this sub-section adopts the joint specimens No. S4 to analyze the shear process due to the most complexity of them in the undulation surface.

**Figure 20:**
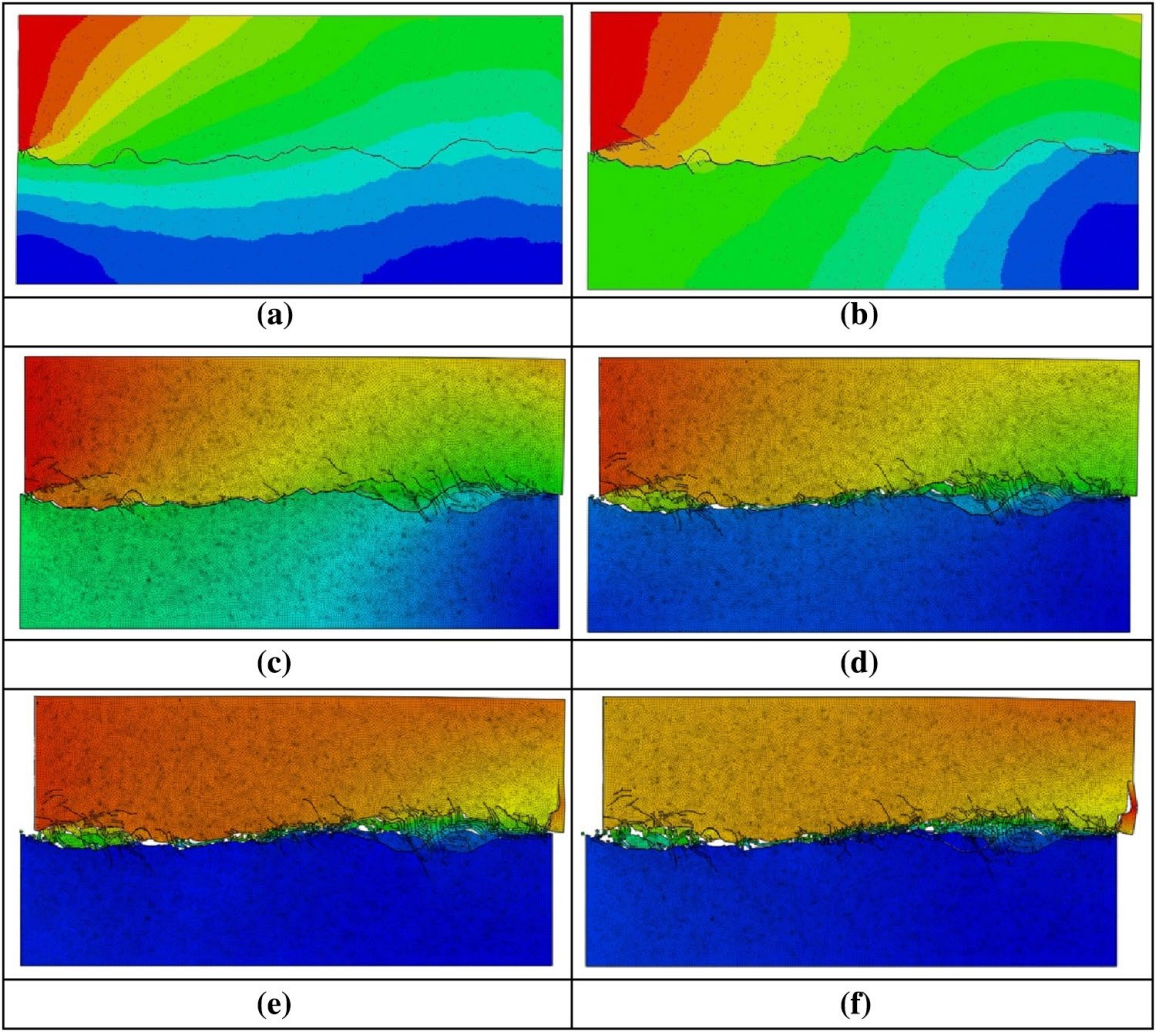
Displacement nephogram of joint model. (**a**) 2 mm. (**b**) 4 mm. (**c**) 6 mm. (**d**) 8 mm. (**e**) 10 mm. (**f**) 12 mm.

In the mesoscopic displacement cloud map during shearing, the increase in displacement of jointed rock mass can be roughly classified into six stages. In the first stage, when the displacement reached 2 mm, the part of the jointed rock mass in contact with the advancing end of the shear box exhibited deformation, which was transmitted from left to right and from top to bottom. The other part of the specimen also exhibited some deformation with the transmission of the joint plane. However, due to the relative displacement of the two parts, a shear crack was produced at the advancing end where the deformation was most obvious (see Fig. 20a). In the second stage, when the displacement reaches 4 mm, the deformation was the largest at the advancing end and the smallest at the distant end, and the deformation is basically the same for most of the specimens. Therefore, the specimens maintained integrity, and only the advancing end and the distant end near the joint plane have local shear cracks due to the large relative displacement (see Fig. 20b). In the third stage, when the displacement reached 6 mm, the displacement of the jointed rock mass is increasing, but the growth rate of deformation in the two parts of the upper and lower joint planes is not the same, and the deformation in the upper joint plane, where the advancing end is located, grows faster than that in the lower part. Shear and tensile cracks appeared around the protrusion, and the cracks at the advancing end continued to develop, and at this moment the jointed rock mass underwent “shear swelling” (see Fig. 20c). In the fourth stage, when the displacement reached 8 mm, the relative displacement between the two parts of the joint plane was getting larger, the number of shear cracks increased obviously, and the specimen was damaged by local fracture. The joint plane protrusion was sheared off, and part of the rock masses was crushed and local dislodgment occurred, marking the entering into the plastic stage. With the increase of shear and tensile cracks, the specimens locally began to gradually fail (see Fig. 20d). In the fifth stage, when the displacement reached 10 mm, the shear and tensile cracks of the specimens were gradually extended to the interior of the rock masses, more rock masses were crushed and compacted under pressure, the roughness was reduced, and the deformation amount of both the upper and the lower jointed rock mass specimen began to get smaller gradually (see Fig. 20e). In the sixth stage, when the displacement reached 12 mm, the shear damage of the specimens were basically completed, the destruction and compaction process of the rock masses reached a certain degree of stability, and the shear crack basically stopped expanding. However, the tensile crack will be gradually expanded to penetration under the action of normal stress (see Fig. 20f).

From the above analysis, it can be seen that the deformation at the advancing end is the largest, which is transmitted to the lower part of the specimens through the joint undulation surface, but the transmission efficiency is obviously not as good as that inside the continuous medium. Therefore, as the deformation increases, the relative displacement between the upper and lower portions of the joint plane becomes more pronounced, leading to the shearing failure of the specimen’s joint plane. When the joint plane exhibits brittle damage, the deformation can only be transmitted to the lower part of the specimens by friction between the specimens, thus the deformation gradually becomes smaller.

**Figure 21:**
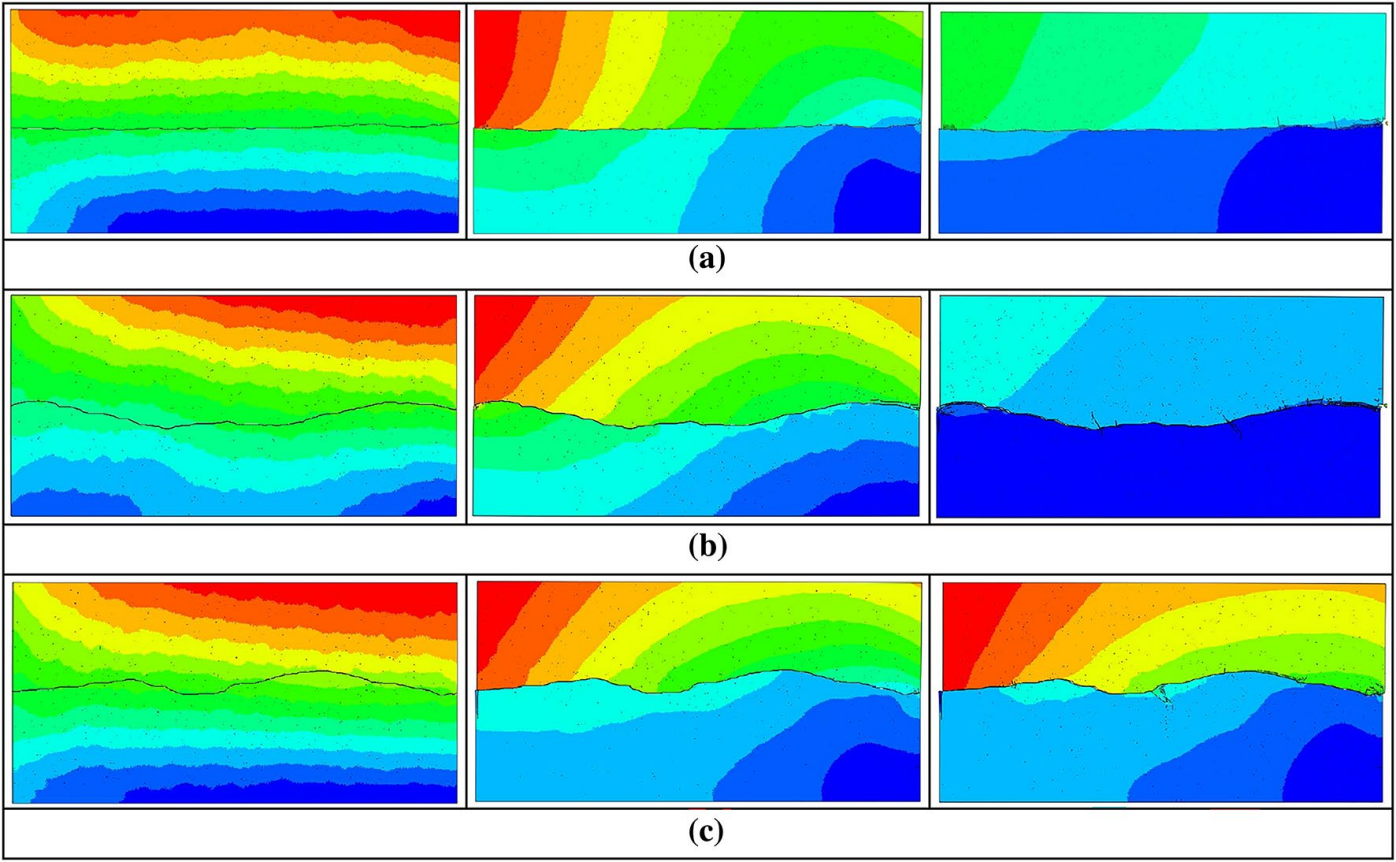
Displacement nephogram of Joints Nos. S1-S3. (**a**) Joint No. S1. (**b**) Joint No. S2. (**c**) Joint No. S3.

Figure 21 shows the mesoscopic displacement change process of Nos. S1-S3 joint specimens under the same confining pressure and shear rate. The comparison of the damages of them with the same displacement is shown as follows:

From the above Fig. 21, it can be seen that as the roughness of the joint planes increases, the reduction of the displacement difference between the joint planes becomes slower. When the displacement difference within the joint plane with smaller roughness decreases gradually, the specimens with larger roughness still had large displacement difference due to the presence of undulating surface protrusions. In the shear process, the existence of high displacement difference makes the joint plane damage for a longer time, and the degree of destruction will be more complete. Therefore, in the actual construction process, the higher roughness of the jointed rock masses has a higher chance of failure damage.

## Discussions on the test and errors

### Discussion on lab compression and shear tests

In this study, direct shear tests on rock-like material specimens were conducted in lab to investigate the shear behavior of jointed rock mass under different shear conditions. The study findings indicate that the peak shear strength of jointed specimens increases with an increase of roughness, but the rate of increase gradually diminishes. This aligns with the conclusions drawn by previous studies such as Han et al.^48^, Wang et al.^49^, and Wu et al.^51^, which explored the impact of joint roughness on stress, displacement evolution, and failure modes under various conditions. The analysis suggests that as roughness increases, the contact area at joint protrusions grows, resulting in higher frictional resistance and hindering relative movement of joint planes during shear. However, when joint specimens reach a certain level of roughness, an increased number of protrusions lead to shear failure involving small protrusions, preventing a rapid increase in peak shear strength. Furthermore, this study reveals a negative correlation between the peak shear strength of jointed specimens and the shear rate, and a positive correlation between it and the confining pressure. This conclusion is consistent with findings made by He et al.^52^, Jiang et al.^54^, Chen et al.^56^, Zhu et al.^57^. This can be explained by that the increased shear rate reduces the time for sufficient contact between joint planes, resulting in reduced frictional resistance. Consequently, the actual contact area at joint contact points decreases, leading to a decrease in peak shear strength. With increased confining pressure, the interlocking of joint planes is strengthened, causing more breakage or crushing of protrusions on joint planes. These consistent observations with previous studies enhance the credibility of the conclusions drawn in this study.

### Discussion on numerical simulation tests

In the course of lab tests, the initiation and propagation of internal specimen failure, as well as the occurrence and progression of surface spalling and cracks, are greatly difficult to be observed. These limitations, often confined by the test apparatus, hinder a clear and timely documentation of these phenomena. This study addressed these challenges by utilizing the ABAQUS, employing the method of global embedding of zero-thickness cohesive elements to accurately replicate the shear process of jointed rock mass with various roughness. The observations made in this study during the evolution of subtle fractures in the jointed rock closely resemble those found by Huan et al.^58^, Bahaaddini et al.^59^, Meng et al.^60^, which used discrete element software PFC to observe microcrack propagation during shear. Taking Bahaaddini’s study^59^ as an example, in which they investigated the degradation of roughness during shear and its impact on jointed rock shear behavior, in the pre-yielding stage, the model only generated small cracks, and when the shear stress reached the yielding shear stress, tensile cracks appeared at the protrusions. The extension of tensile cracks and the fragmentation of cracks led to a decrease in shear stress to residual stress, accompanied by a rapid increase in the number of cracks. This aligns with the observations in this study, where the process of crack initiation, extension, and eventual penetration into the interior was consistent with the initial stages of crack development observed in displacement cloud maps. However, rock is not a purely discontinuous medium, and an improved finite element model can equally provide a robust simulation of the shear process. The work of Han et al.^62^, employing finite element methods to categorize shear processes into four typical stages, is well consistent with the six more detailed shear failure processes observed in numerical simulations in this study. The studies by these scholars validate the authenticity and superiority of the numerical simulation works in this study.

### Research error analysis

This study explored the impact of roughness on the shear performance of jointed rock-like specimens, demonstrating phenomena consistent with the elastic stage observed by scholars like Han et al.^48^, Wang et al.^49^, and Wu et al.^51^. However, in this study, a slight abrupt change in shear stress was observed with increasing shear displacement. The analysis suggests that this abrupt change might be attributed to the influence of large roughness on some joints and operational errors inevitable in specimen preparation, leading to minor local damage during the shear test of jointed rock. After completing the laboratory tests, numerical simulations can further verify the correctness of the test results. The numerical simulations by Huan et al.^58^, Bahaaddini et al.^59^, Meng et al.^60^, and Han et al.^62^ show a good consistence with test data. However, the simulated data in this tests show some deviations from the displacement-stress curve obtained in the lab tests. The analysis suggests that in establishing the numerical model for this study, the assumption that the upper and lower joint planes are tightly bonded led to the absence of the initial densification stage detected in the tests. Despite efforts to closely match the simulation to test results, the test specimens were not prepared under ideal conditions. While the numerical simulation considered the influence of roughness variations on shear strength under direct shear, the joint planes in the numerical model were assumed to be smooth, whereas the surfaces of the test specimens were relatively rough due to material characteristics and lacked smoothing treatment, resulting in simulated results slightly lower than test results.

## Conclusion

In this study, compressive shear experiments were conducted on rock-like material specimens by means of lab tests to investigate the shear performance of jointed rock mass specimens under different shear conditions. Through the combination of laboratory and numerical simulation, we investigated the evolution mechanism of mesoscopic displacement of jointed rock mass under shear loading and the damage characteristics of jointed rock mass with different roughness. The main conclusions are as follows:

- (i) By controlling and adjusting the shear variables of roughness, shear rate, and confining pressure while keeping other conditions the same, we conducted the investigation, which reveals that the shear strength of the specimens increases with the increase of roughness, decreases with the increase of shear rate, and increases with the increase of the confining pressure.
- (ii) During the shear process, jointed rock mass specimens undergo brittle damage, and cracks and spalling are generated from the joint planes to both sides. The distribution of cracks is greatly affected by the roughness of the joint plane. When the roughness of the jointed rock mass is small, the joint plane is relatively smooth, and the distribution of cracks is more uniform; when the roughness is larger, the distribution of cracks is more concentrated because of the larger undulation, and the cracks are mainly concentrated in the protruding part of the joint plane.
- (iii) Normalized peak shear strength under different conditions demonstrate that the shear strength of the specimen part with smaller roughness grows faster. This is due to that when the roughness reaches a certain degree, more protrusions are generated, and shear damage with small protrusions occur, hindering the peak shear strength to further grow fast. Similarly, it can be observed from the normalization of the peak shear strength that a slower shear rate has a greater impact on the peak, and when the shear rate reaches a certain value, the effect of the shear rate on the peak shear strength becomes less obvious.
- (iv) The evolution mechanism of mesoscopic displacement of jointed rock mass when subjected to shear was analyzed using ABAQUS. It is found that the joint plane with higher roughness has a larger difference in mesoscopic displacement inside the joint plane due to more surface protrusions, which leads to faster damage and higher degree of damage in the specimens with higher roughness. Therefore, in the actual construction process, high-roughness joints should be prioritized for timely reinforcement to prevent sudden failure damage.

## Data Availability

The datasets generated during and analyzed during the current study are available from the corresponding author (Feng Jiang) on reasonable request.
